# Genetically engineered nano‐melittin vesicles for multimodal synergetic cancer therapy

**DOI:** 10.1002/btm2.10482

**Published:** 2023-01-04

**Authors:** Jianzhong Zhang, Xue Liu, Yutian Xia, Shuyu Xu, Xuan Liu, Haiqing Xiao, Xiaoyong Wang, Chao Liu, Gang Liu

**Affiliations:** ^1^ State Key Laboratory of Molecular Vaccinology and Molecular Diagnostics, School of Public Health Xiamen University Xiamen China; ^2^ Center for Molecular Imaging and Translational Medicine, School of Public Health Xiamen University Xiamen China; ^3^ National Institute of Diagnostics and Vaccine Development in Infectious Diseases, School of Public Health Xiamen University Xiamen China; ^4^ State Key Laboratory of Cellular Stress Biology, Innovation Center for Cell Biology, School of Life Sciences Xiamen University Xiamen China

**Keywords:** Cancer therapy, Drug delivery, Melittin, Membrane vesicles, Nanomedicine

## Abstract

Melittin, the principal constituent in bee venom, is an attractive candidate for cancer therapy. However, its clinical applications are limited by hemolysis, nonspecific cytotoxicity, and rapid metabolism. Herein, a novel genetically engineered vesicular antibody‐melittin (VAM) drug delivery platform was proposed and validated for targeted cancer combination therapy. VAM generated from the cellular plasma membrane was bio‐synthetically fabricated, with the recombinant protein (hGC33 scFv‐melittin) being harbored and displayed on the cell membrane. The bioactive and targetable nanomelittin conjugated by hGC33 scFv could be released in an MMP14‐responsive manner at tumor sites, which reduced off‐target toxicity, especially the hemolytic activity of melittin. Importantly, VAM could be loaded with small‐molecule drugs or nanoparticles for combination therapy. Nanomelittin formed pores in membranes and disturbed phospholipid bilayers, which allowed the anticancer agents (i.e., chemotherapeutic drug doxorubicin and sonosensitizer purpurin 18 nanoparticles) co‐delivered by VAM to penetrate deeper tumor sites, leading to synergistic therapeutic effects. In particular, the punching effect generated by sonodynamic therapy further improved the immunomodulatory effect of nanomelittin to activate the immune response. Taken together, our findings indicate that clinically translatable VAM‐based strategies represent a universal, promising approach to multimodal synergetic cancer therapy.

## 1 INTRODUCTION

Cancer therapy has stepped into the era of multidisciplinary treatment (MDT),^1^ calling for rational and orchestrating combination strategies.^2^, ^3^ Traditional therapeutic modalities have unavoidable limitations, such as chemotherapy‐associated drug resistance,^4^ and eventually, result in tumor survival and poor prognosis. To achieve satisfactory treatment effects, a more effective class of technologies for cancer treatment is required.

**SCHEME 1:**
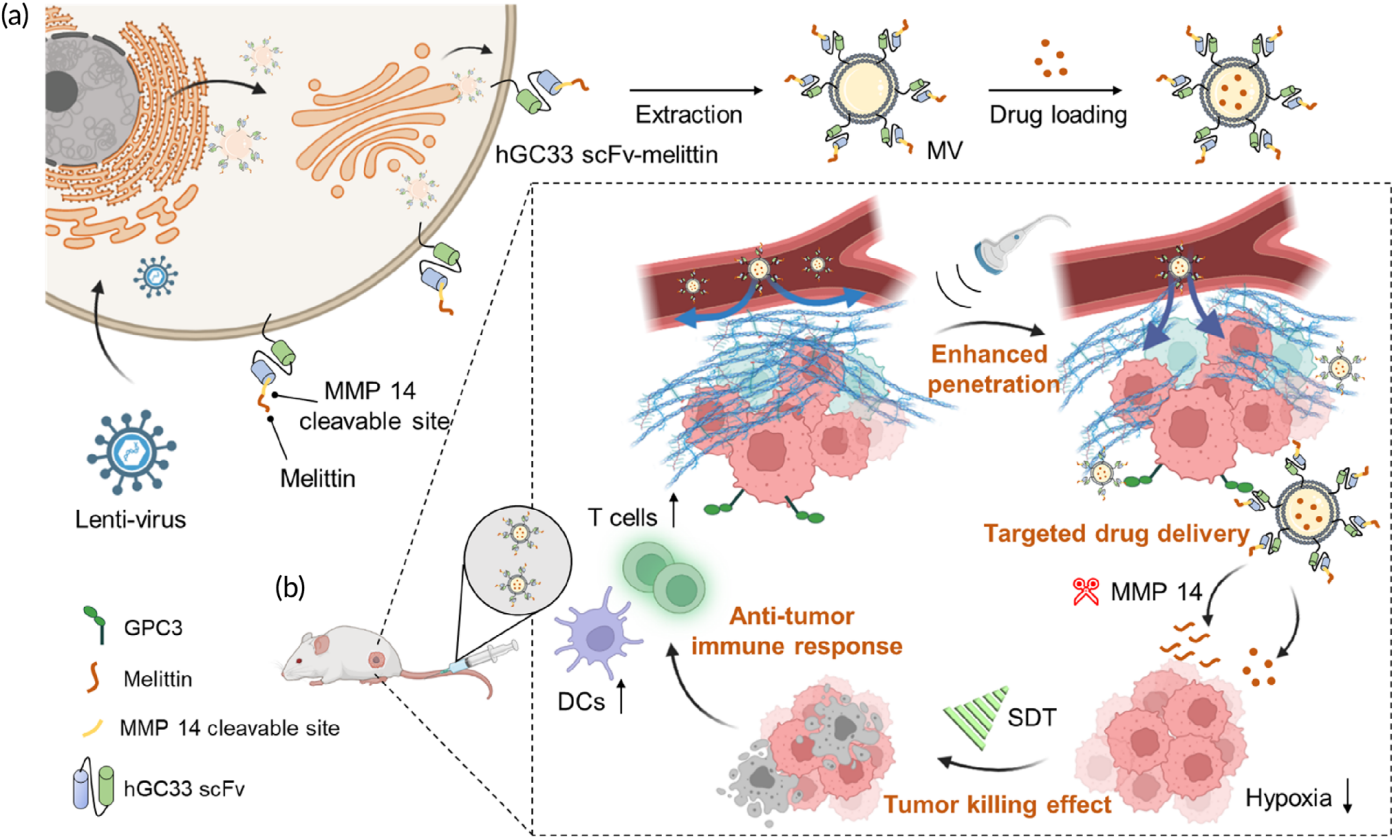
Designing VAM for targeted cancer combination therapy. (a) Vesicular antibody‐melittin (VAM) was constructed by a genetic engineering procedure. As a synergistic drug delivery platform, VAM could be delivered to GPC3‐positive tumor cells, which reduced off‐target toxicity; hGC33 scFv‐melittin MVs could be loaded with sonosensitizers and synergize with SDT. (b) The bioactive nanomelittin could be released in an MMP14‐responsive manner at tumor sites. The enhanced accumulation and penetration of drugs in tumors were achieved owing to membrane pore formation and sonoporation. The punching effect generated by SDT further improved the immunomodulatory effect of nanomelittin to activate the antitumor immune response.

In recent years, cytotoxic peptides have been applied in cancer treatment due to their low immunogenicity, good biocompatibility, and decreased likelihood of developing drug resistance.^5^ Among these, melittin,^6^, ^7^ the primary bioactive component of bee venom, has attracted considerable interest for its antitumor properties. It achieves these effects by disrupting cell membranes physically and chemically, thereby altering cells' permeability.^8^ Furthermore, to some degree, melittin exhibits immunoregulatory effects,^9^, ^10^ which suggests it may be capable of modulating the suppressive immune microenvironment characteristic of solid tumors. Nevertheless, its severe side effects (e.g., hemolysis) when injected intravenously and nonspecific lytic activity have restricted melittin's clinical application.^11^ Targeted therapy,^12^, ^13^ enables delivering therapeutic agents to specific tumor sites and avoiding healthy tissues; as such, this represents a robust therapeutic approach to tumor management that confers specificity to otherwise non‐specific modalities. In the meantime, monotherapy commonly failed to meet clinical needs due to limited therapeutic efficacy, drug‐loaded nanoarchitectures integrating functional components demonstrated favorable synergistic therapeutic effects,^14^, ^15^ and new strategies are waiting to be excavated for efficient therapy modalities. For example, cellular membrane vesicles (MVs),^16^, ^17^ are emerging drug delivery carriers that originate from the cellular plasma membrane and possess excellent biocompatibility and plasticity. MVs could help actualize drug encapsulation and genetic engineering technologies,^18^, ^19^, ^20^ thus opening new frontiers for next‐generation therapeutics. We hypothesized that a genetically engineered vesicular antibody‐melittin (VAM) drug delivery nanoplatform for targeted cancer combination therapy could be developed that addresses the limitations of melittin. As a proof of principle, we designed genetically fused hGC33 scFv‐melittin anchored on cellular MVs targeted to GPC3‐positive hepatocellular carcinoma (HCC; Scheme 1).

For target selection, we selected the highly specific glypican‐3 (GPC3)^21^, ^22^, ^23^ cell surface protein expressed in 70%–80% of HCC cases as the target antigen. As a precisely characterized HCC‐associated antigen, human GPC3 reliably distinguishes HCC from healthy tissues and is, therefore, recognized as a promising treatment target for HCC. hGC33 (humanized GC33),^24^, ^25^ a potential antitumor agent for HCC, was identified as the ideal antibody for human GPC3. Traditionally, chemotherapeutic drugs were chosen for their cytotoxic payloads and conjugated by a complicated chemical modification process. Particularly, heterogeneity caused by random chemical conjugation hinders the clinical application of this formulation. For bench‐to‐bedside translation, the conjugation processes are supposed to produce scalable and homogeneous batches. When compared with conventional chemical modification,^26^, ^27^ genetic fusion possesses the advantages of generating homogeneous mixtures and stable conjugation, which are crucial for an optimized regimen. We genetically fused hGC33 scFv with melittin via an MMP14‐responsive linker^28^ and displayed fusion protein on the membrane surfaces of mammalian cells (BHK‐21). Remarkably, the clinical applications of targeted therapy have been largely restricted to hematological malignancies and generally failed in solid tumors owing to their macromolecular structure.^29^ The conjugated nanomelittin with membrane‐disrupting function may pave the way for this well‐coordinated design against solid malignancies.

To verify VAM as a versatile drug delivery platform and design a synergistic therapeutic regimen, the chemotherapeutic drug doxorubicin (DOX) was encapsulated into MVs (denoted as DOX@control MVs or DOX@hGC33 scFv‐melittin MVs). Because hGC33 scFv MVs loaded with DOX target GPC3‐positive tumor cells specifically, thus reducing the dose of this small molecule drug. Though applicated in clinics extensively for several years, lacking selectivity renders DOX resulting in severe side effects such as myelosuppression or cardiotoxicity,^30^, ^31^ and high doses are needed to ensure the sufficient accumulation in tumor sites as to reach lethal concentration. Harnessing VAM as a targeted delivery carrier may bring beneficial effects. Besides chemotherapy, sonodynamic therapy (SDT),^32^, ^33^, ^34^ an emerging noninvasive technique, was another therapeutic modality choice coupling with VAM we designed. Low‐intensity ultrasound (US) activates the sonosensitizer to kill tumor cells in a reactive oxygen species‐dependent manner.^32^, ^35^ Owing to its high penetration depth and cavitation effect, US was widely used for biomedical applications.^36^, ^37^ Sonosensitizer (Purpurin 18 nanoparticles, termed nP18) loaded with hGC33 scFv‐melittin MVs could be delivered to tumor sites specifically and synergizes with an ultrasound focused precisely on tumor sites, the selective killing of GPC3‐positive tumor cells without damaging healthy tissues was achieved. We hypothesized that hGC33 scFv‐melittin MVs combined with SDT have two potential advantages for eliciting substantial antitumor effects. First, sonoporation generated by SDT could synergize with the pore‐forming effect of melittin, thereby enhancing the penetration of the co‐delivered drugs. Second, tumor‐infiltrating lymphocytes (TILs) in the tumor microenvironment (TME) play a vital role in the tumor environment,^38^, ^39^ and accompanied by the increase of TILs due to loosened tumor tissue, melittin could exert its immunomodulatory effect. We evaluated the ability of nP18@hGC33 scFv‐melittin MVs to eradicate tumors and reshape the immune microenvironment. Remarkably, VAM avoids the generation of severe side effects through intravenous administration. Both DOX@hGC33 scFv‐melittin MVs and nP18@hGC33 scFv‐melittin MVs exhibited significant antitumor effects with biological safety. It is believed that VAM could serve as a promising platform for targeted cancer combination therapy.

## 2 RESULTS

### 2.1 Preparation and characterization of hGC33 scFv‐melittin MVs

**FIGURE 1:**
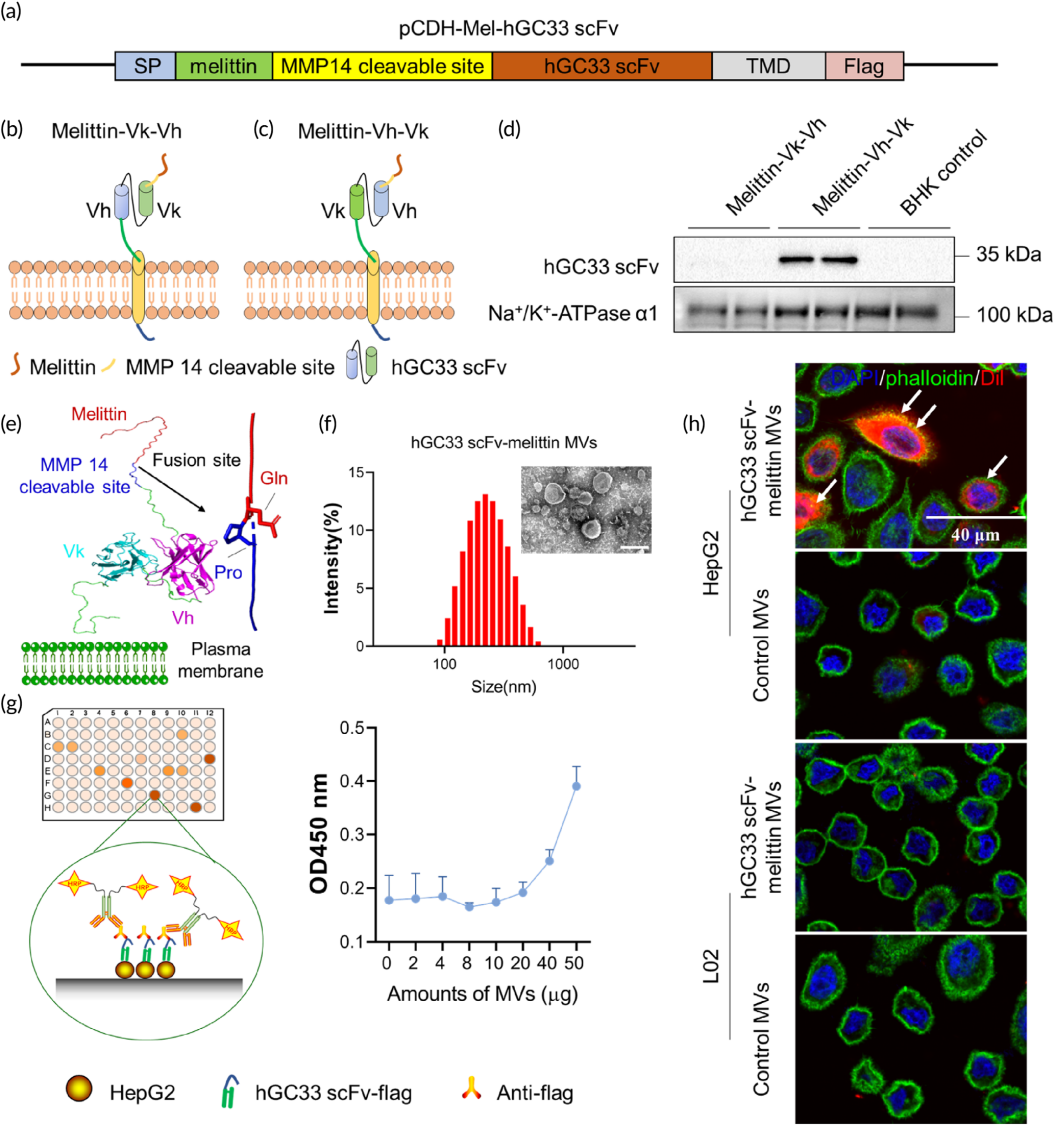
Construction and characterization of hGC33 scFv‐melittin membrane vesicles (MVs). (a) Schematic representation of recombinant hGC33 scFv‐melittin protein. Guided by the signal peptide (SP), the recombinant protein was anchored on the cell membrane surface; TMD, transmembrane domain. (b,c) Schematic illustration of the relative spatial position between the membrane and hGC33 scFv. Vh, variable heavy chain; Vk, variable light chain. (d) Western blotting analysis of hGC33 scFv from extracted MVs. (e) Three‐dimensional structure simulation of hGC33 scFv‐melittin (Melittin‐Vh‐Vk). The image was generated in Modeler 10.3 software. (f) Morphology images of MVs by transmission electron microscopy; Size distribution of MVs by dynamic light scattering. The average diameter is 225.7 nm. (g) Cell‐based enzyme‐linked immunosorbent assay for binding ability verification of hGC33 scFv and GPC3. (h) The targeting ability verification by laser scanning confocal microscopy. Blue, DAPI. Green, phalloidin. Red, DiI. Scale bar: 40 μm.

hGC33 scFv‐melittin fusion protein anchored on the membrane surface (Figure 1a) was constructed using the genetically engineered method reported previously.^40^ Briefly, recombinant hGC33 scFv‐melittin genes were constructed by sequentially fusing HA signal peptide (harboring plasma membrane), melittin, MMP14 responsive linker, and hGC33 scFv genes, which form the components of the outer membrane. There are two cases of spatial position between the outer membrane and variable chains of single‐chain variable fragment (scFv; Figure 1b,c) that may influence the structure and function of scFv, so we designed both. After continuous passage and puromycin screening, stable cell lines were obtained (Figures S1 and S2), and hGC33 scFv (Melittin‐Vh‐Vk) was detected by western blotting (Figure 1d) and immunofluorescence (Figures S3 and S4). Notably, only melittin‐Vh‐Vk could be detected, so we chose Melittin‐Vh‐Vk as hGC33 scFv‐melittin for the following assays. The 3D structure of Melittin‐Vh‐Vk was simulated through homology modeling (Figure 1e). The MVs were prepared through multistep density gradient ultracentrifugation,^19^ the morphology and average diameter of MVs were characterized by transmission electron microscopy (TEM), scanning electron microscopy, and dynamic light scattering (DLS), respectively (Figures 1f, S5 and S6). The extracted MVs exhibited good stability while stored at 4°C in PBS or serum for several days (Figure S7). The potential of MVs was analyzed by DLS (Figure S8). Cell‐based enzyme‐linked immunosorbent assay (ELISA) and cellular uptake assay indicated that hGC33 scFv‐melittin MVs were specifically bound to GPC3‐positive HepG2 cells (Figure 1g,h). These results demonstrated that the construction of VAM was successful.

### 2.2 Functional evaluation of hGC33 scFv‐melittin MVs *in vitro*

**FIGURE 2:**
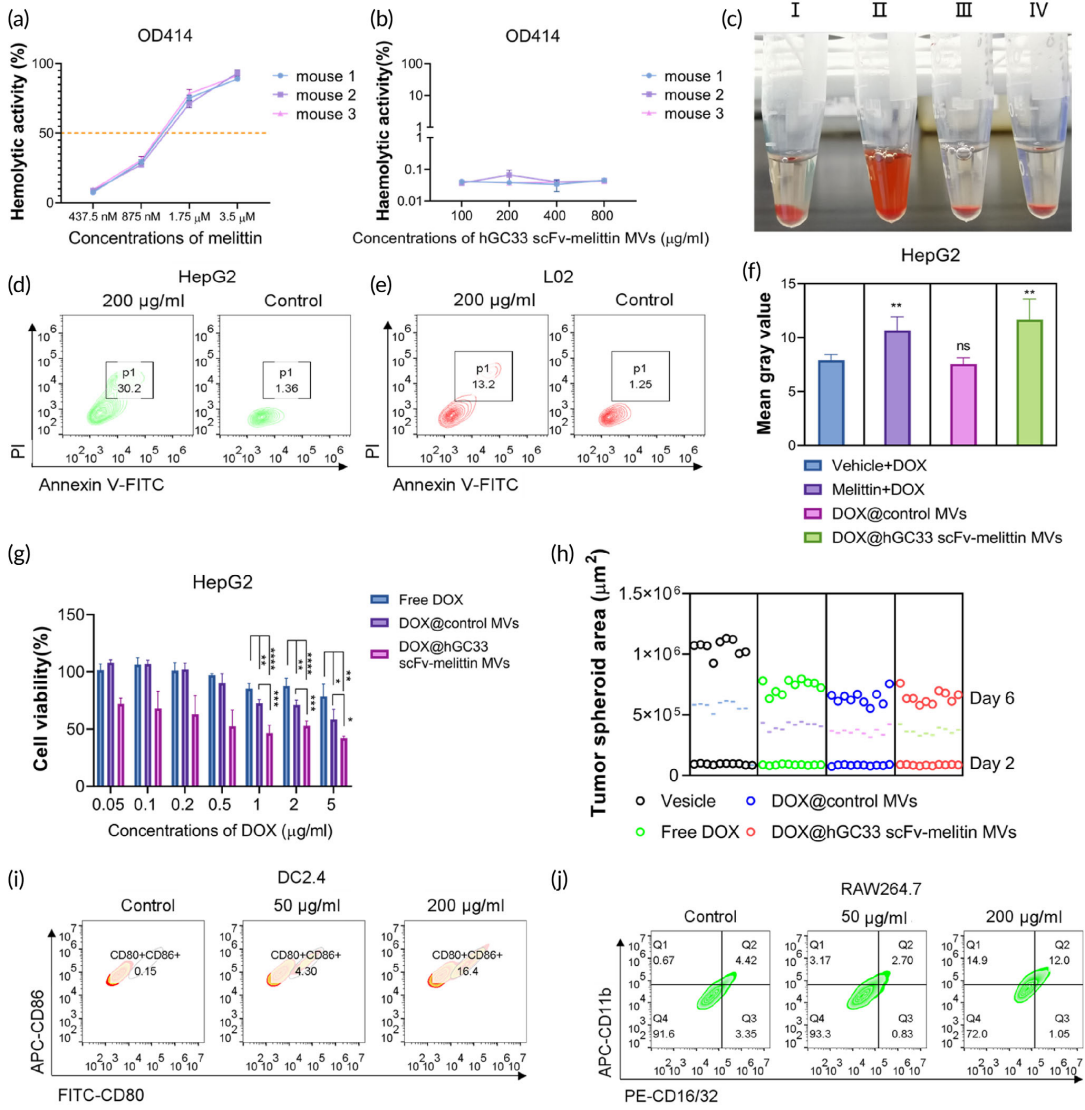
Functional evaluation of hGC33 scFv‐melittin membrane vesicles (MVs) *in vitro*. (a) Hemolytic activity of melittin. (b) Hemolytic activity of hGC33 scFv‐melittin MVs. (c) Hemolysis is caused by melittin. I: Saline, II, Melittin, III, hGC33 scFv‐melittin MVs, IV, Control MVs. (d) and (e) Apoptosis experiment of hGC33 scFv‐melittin MVs in HepG2 and L02 cells, respectively. (f) Uptake of doxorubicin (DOX) in different groups by HepG2 cells. (g) Cell cytotoxicity assay in different groups in HepG2 cells (50 μg control MVs or hGC33 scFv‐melittin MVs were used for DOX encapsulation in each group). (h) Tumor spheroid inhibition assay by DOX in different groups, the surface areas were calculated by Day 2 and day 6 postcell seeding. (i) Immunoregulatory effect of hGC33 scFv‐melittin MVs to DC2.4. (j) Immunoregulatory effect of hGC33 scFv‐melittin MVs to RAW264.7. ns, not significant, *p < 0.05, **p < 0.01, ***p < 0.001, and ****p < 0.0001.

The apoptosis assay showed that “melittin only MVs” had nonselective cytotoxic effect to healthy L02 cells. To focus on the functions of hGC33 scFv‐melittin MVs, “none of those MV” was chosen as a control for the following experiments (Figure S9). Free melittin could cause severe hemolysis (Figures 2a and S10) when coincubated with red blood cells (RBCs) derived from Balb/c mice. When compared with free melittin, hGC33 scFv‐melittin MVs protected RBCs from hemolysis (Figure 2b,c), which revealed the potential of intravenous administration. Furthermore, the tails of the free melittin group showed severe petechia and necrosis, while those of the hGC33 scFv‐melittin MVs group were injured less (Figure S11). Further, we verified the capacity of melittin to induce cell apoptosis (Figures S12 and S13). hGC33 scFv‐melittin MVs showed selectivity in inducing apoptosis between cancer cells and healthy cells (Figures 2d and 2e); this was due to the specific binding between hGC33 scFv and GPC3. Considering that vesicles were favorable drug carriers, leveraging the tumor selectivity of hGC33 scFv‐melittin MVs may realize the efficient delivery of chemotherapeutics. We encapsulated DOX with MVs and tested the uptake of DOX@hGC33 scFv‐melittin MVs (Figures 2f, S14, and S15). DOX@hGC33 scFv‐melittin MVs showed enhanced uptake by HepG2 cells, thus indicating the more potent cytotoxicity of DOX@hGC33 scFv‐melittin MVs. In a cell cytotoxicity test (Figures 2g and S16), DOX@hGC33 scFv‐melittin MVs exhibited enhanced cytotoxicity compared with free DOX. Scratch assay showed that DOX@hGC33 scFv‐melittin MVs inhibited cell mobility in a time‐dependent manner (Figure S17). On the one hand, the element hGC33 scFv conferred hGC33 scFv‐melittin MVs targeting ability. On the other hand, the element melittin conferred hGC33 scFv‐melittin MVs toxicity. Moreover, we constructed a multicellular tumor spheroid model to confirm the enhanced cytotoxicity of DOX@hGC33 scFv‐melittin MVs (Figures 2h and S18). We next evaluated the immunoregulatory effect of melittin under low‐toxicity concentrations (Figures S19 and S20). The data showed that free melittin hardly upregulates the expression of co‐stimulatory molecules of DC2.4 or RAW264.7. In contrast, melittin anchored to the membrane, hGC33 scFv‐melittin, activated DC2.4 and RAW264.7 in a concentration‐dependent manner (Figure 2i,j). The data above suggested the potential of hGC33 scFv‐melittin MVs acted as a carrier platform for drug delivery. Meanwhile, the engineered VAM could be harnessed to modulate the immunosuppressive TME.

### 2.3 Enhanced drug penetration of hGC33 scFv‐melittin MVs in 3D tumor spheroid and *ex vivo* model

**FIGURE 3:**
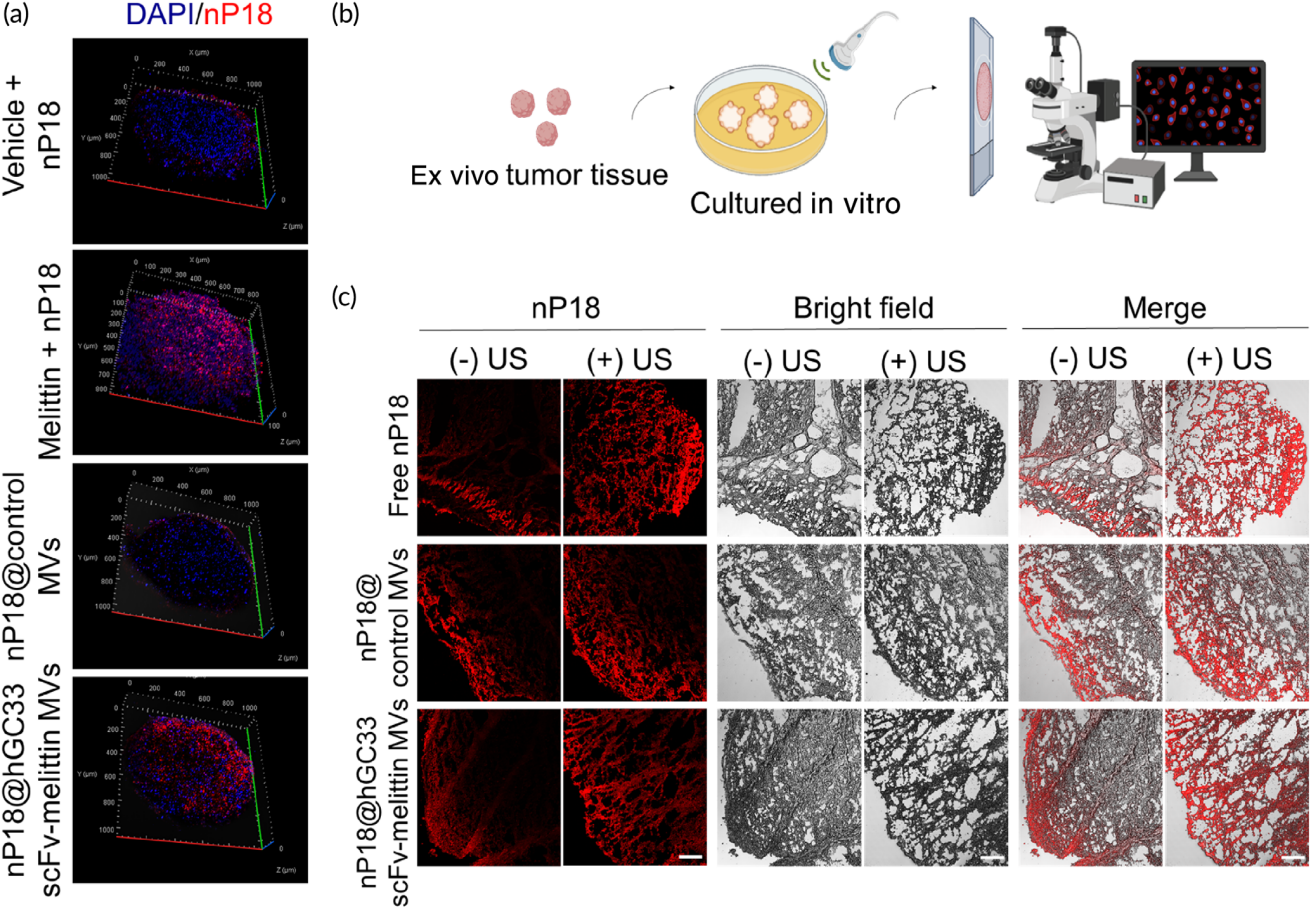
Enhanced drug penetration of hGC33 scFv‐melittin membrane vesicles (MVs). (a) Enhanced nP18 penetration in 3D tumor spheroid model. Blue, DAPI; Red, nP18. (b) and (c) Enhanced nP18 penetration in *ex vivo* tumor tissue model. Red, nP18. Scale bar: 200 μm.

Subsequently, to characterize the penetration capacity of hGC33 scFv‐melittin MVs in solid tumors, a nanosonosensitizer (nP18)^41^ was encapsulated by hGC33 scFv‐melittin MVs. We first studied the cellular uptake of nP18 by HepG2 in a 2D cell culture system (Figure S21). Encouraged by the enhanced uptake in the nP18@hGC33 scFv‐melittin MVs group, 3D tumor spheroid (multicellular tumor spheroid [MCTS]) was designed to mimic the native conditions of solid tumors and confirm the efficient tumor penetration (Figures 3a and S22). When compared with the free melittin group, hGC33 scFv‐melittin MVs retained the ability of melittin to disrupt the membrane, and nP18@hGC33 scFv‐melittin MVs could diffuse into the inner core of the MCTS from the surface to the middle. In contrast, the fluorescence signals of the free nP18 and nP18@control MVs groups were only displayed on the periphery of the spheroids. Moreover, the mechanical perturbation induced by US‐mediated cavitation could further enhance the penetration of nanodrugs. Dissected tumor tissues were prepared to validate the ability of hGC33 scFv‐melittin MVs to enhance drug penetration *ex vivo* (Figure 3b). Ultrasonic waves dramatically enhanced the penetration of nP18 (Figure 3c), which greatly improved the drug accumulation and penetration for *in vivo* applications of this combination strategy.

### 2.4 *In vivo* biodistribution of hGC33 scFv‐melittin MVs

**FIGURE 4:**
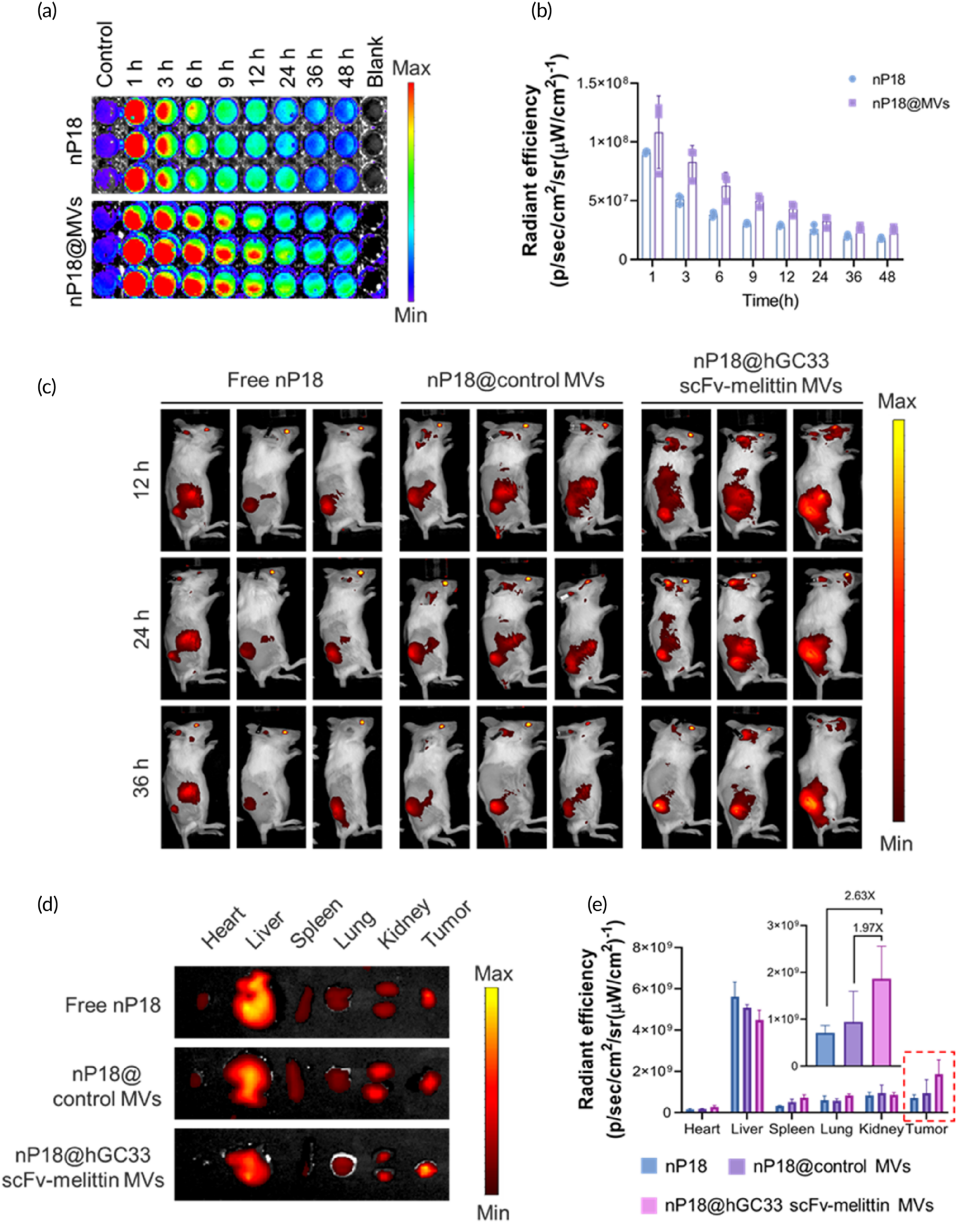
Biodistribution of nP18 and nP18@membrane vesicles (MVs). (a) Retention time in the blood of free nP18 and nP18@MVs and (b) Radiant efficacy of nP18 in blood at different time points. (c) *In vivo* fluorescence imaging of H22‐GPC3 tumors in mice upon injection of free nP18, nP18@control MVs and nP18@hGC33 scFv‐melittin MVs intravenously after 12, 24, and 36 h. (d) *Ex vivo* fluorescence imaging and (e) Relative fluorescence intensity of tumors and major organs after 36 h of injection (*n* = 3).

To evaluate the targeting and accumulation ability of hGC33 scFv‐melittin MVs, nP18 was employed as a carrier for visualization owing to its optical imaging property of fluorescence. After being injected intravenously, peripheral blood was collected at 1, 3, 6, 9, 12, 24, 36, and 48 h, and the fluorescence intensity was detected by *in vivo* imaging system (IVIS; Figure 4a), compared with the free nP18 group, nP18 encapsulated in MVs (nP18@MVs) exhibited longer blood circulation time (Figure 4b). MV‐camouflaged NPs achieved long‐term circulation in the blood of animals. Prolonged blood circulation increases the stochastic probability of the accumulation of the loaded drugs into tumor sites. To explore the targeted delivery ability of hGC33 scFv‐melittin MVs to GPC3‐positive tumor cells *in vivo*, we monitored the biodistribution of nP18 at 12, 24, and 36 h postintravenous injection by fluorescence imaging. The fluorescence intensity of the tumor region appeared to be time‐dependent among the different groups (Figure 4c). Compared with the free nP18 and nP18@control MVs, nP18@hGC33 scFv‐melittin MVs had higher fluorescent signals, suggesting more drug accumulated in the tumor region. After 36 h post‐injection, the tumor tissues and major organs were harvested for *ex vivo* fluorescence imaging (Figure 4d). By semiquantitative analysis based on *ex vivo* fluorescent imaging, the nP18@hGC33 scFv‐melittin MVs group had more nP18 accumulation in the tumor region compared to free nP18 (2.63 times) and nP18@control MVs (1.97 times) groups (Figure 4e). These data demonstrated that nP18@hGC33 scFv‐melittin MVs could efficiently target, penetrate, and accumulate in tumor regions. To further verify the enhanced accumulation of nanomaterials in tumor tissues from the nP18@hGC33 scFv‐melittin MVs group, frozen tumor slices isolated from different treatments were imaged according to their P18 fluorescence. Remarkably, more nP18 was found in the nP18@hGC33 scFv‐melittin MVs group (Figure S23). The accumulation and retention appeared to be time‐dependent, and peaked at 24 h post‐injection. Taken together, these data demonstrated the potential of fluorescence‐guided SDT therapy.

### 2.5 Antitumor effect of VAM combined with chemotherapy in HepG2 bearing Balb/c nude mice

**FIGURE 5:**
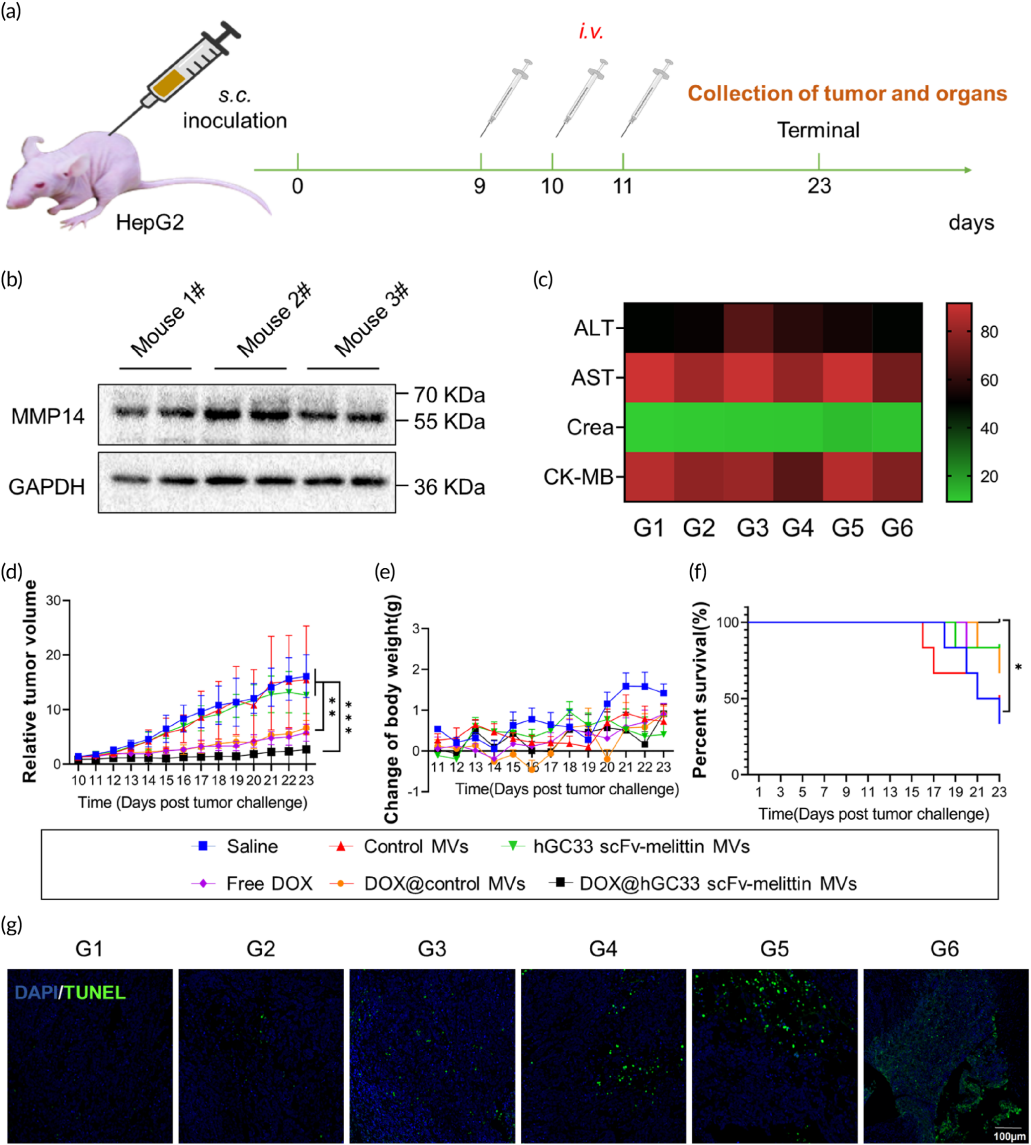
Doxorubicin (DOX)@hGC33 scFv‐melittin membrane vesicles (MVs) showed efficient antitumor efficacy *in vivo*. (a) Timeline of the establishment of the HepG2‐bearing nude mice model (n = 5). (b) Western blotting analysis of MMP14 expressing level in dissected tumor tissues. (c) Blood biochemical parameter. (d) Relative tumor volume of different treatment groups. (e) Change of body weight. (f) Survival rate. (g) TUNEL assay and DAPI staining of dissected tumor tissues in different groups. Blue, DAPI; Green, TUNEL. Scale bar: 100 μm. Group 1, saline; Group 2, control MVs; Group 3, hGC33 scFv‐melittin MVs; Group 4, free DOX; Group 5, DOX@control MVs; Group 6, DOX@hGC33 scFv‐melittin MVs. *p < 0.05, **p < 0.01, and ***p < 0.001.

To evaluate the antitumor ability of DOX@hGC33 scFv‐melittin MVs *in vivo*, a HepG2‐bearing nude mice model was constructed. HepG2 cells were inoculated into the right flank of Balb/c nude mice (Figure 5a), and the mice were randomly divided into six groups: Group 1, saline; Group 2, control MVs; Group 3, hGC33 scFv‐melittin MVs; Group 4, free DOX; Group 5, DOX@control MVs; Group 6, DOX@hGC33 scFv‐melittin MVs. The dosage of DOX was 1 mg/kg (200 μg MVs per mouse per dose). We detected the expression of MMP14 in dissected HepG2 tumor tissues (Figure 5b) as indicative of the MMP14‐responsive release of melittin. During the treatment, blood was collected from each treatment group, and the typical blood biochemical markers, including alanine transaminase (ALT), aspartate aminotransferase (AST), creatinine (CREA), and creatine kinase‐MB (CK‐MB) were quantified. The data confirmed the favorable biosafety of the combination therapy (Figure 5c). The tumor sizes and mice's weights were measured daily (Figures 5d and S24). The data showed that, compared with the saline group, the DOX@hGC33 scFv‐melittin MVs group inhibited the growth of tumors significantly, which may be attributed to the targeted delivery of DOX (Figure S25). The free DOX and DOX@control MVs suppressed tumor growth to some extent, but these were not as significant as the DOX@hGC33 scFv‐melittin MVs group. The body weights recorded from each group showed no obvious fluctuations, indicating the safety of the *in vivo* application of this formulation (Figure 5e). Survival analysis also showed the mice of the DOX@hGC33 scFv‐melittin MVs group had longer lifespans than the other groups (Figure 5f). TUNEL assay was conducted to detect the apoptosis of tumors (Figure 5g), and the DOX@hGC33 scFv‐melittin MVs group had a higher degree of apoptosis. Moreover, major organs, including the heart, liver, spleen, lung, and kidney from each group, were stained by H&E, and no obvious side effects were found from the combination therapy (Figure S26). These results demonstrate that VAM combined with chemotherapy effectively suppressed tumor development.

### 2.6 Antitumor effect of VAM combined with SDT in H22‐GPC3 bearing Balb/c immune‐competent mice

**FIGURE 6:**
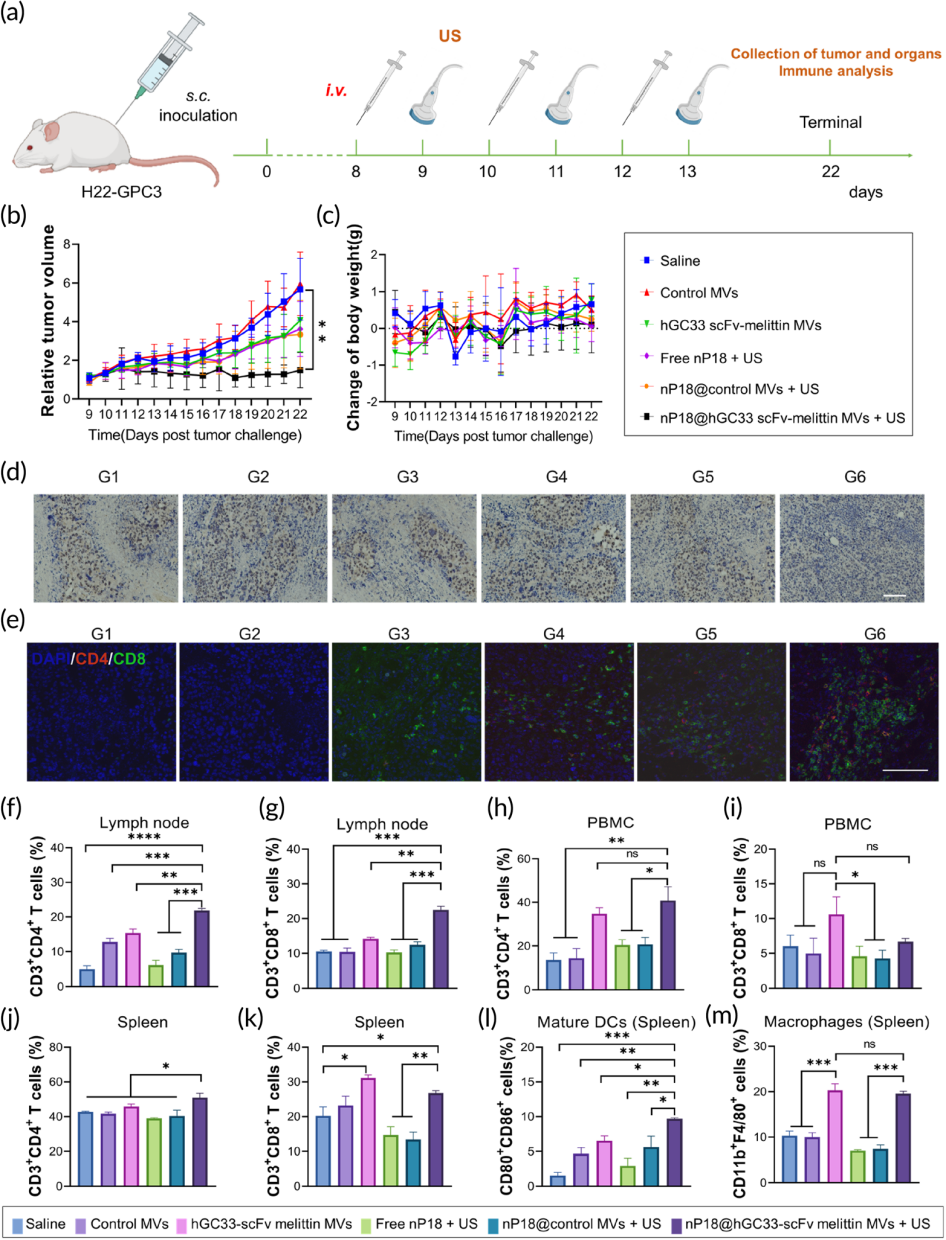
nP18@hGC33 scFv‐melittin membrane vesicles (MVs) showed efficient antitumor efficacy *in vivo*. (a) Timeline of the establishment of the H22‐GPC3 bearing Balb/c mice model (*n* = 6). (b) Relative tumor volume of different treatment groups. (c) Change of body weight. (d) Micrograph images of tumor slices with hypoxia‐inducible factor (HIF‐1α) immunohistochemical staining of different treatment groups. Scale bar: 100 μm. (e) CD4^+^/CD8^+^ T cells staining of tumors with different treatments. Scale bar: 100 μm. (f) The population of CD3^+^CD4^+^ T cells in the lymph node. (g) The population of CD3^+^CD8^+^ T cells in the lymph node. (h) The population of CD3^+^CD4^+^ T cells in the PBMC. (i) The population of CD3^+^CD8^+^ T cells in the PBMC. (j) The population of CD3^+^CD4^+^ T cells in the spleen. (k) The population of CD3^+^CD8^+^ T cells in the spleen. (l) The population of CD80^+^CD86^+^ DCs in the spleen. (m) The population of CD11b^+^F4/80^+^ macrophages in the spleen. Group 1, saline; Group 2, control MVs; Group 3, hGC33 scFv‐melittin MVs; Group 4, free nP18 + ultrasound (US); Group 5, nP18@control MVs + US; Group 6, nP18@ hGC33 scFv‐melittin MVs + US. ns, not significant; *p < 0.05, **p < 0.01, ***p < 0.001, and ****p < 0.0001.

To evaluate the *in vivo* antitumor efficacy of VAM combined with SDT and validate the immunoregulatory effect of hGC33 scFv‐melittin, we inoculated GPC3 overexpressing H22 cells (H22‐GPC3) constructed by our group previously on the right flank of Balb/c immune‐competent mice (Figure 6a). H22‐GPC3‐bearing Balb/c mice were randomly divided into six groups: Group 1, saline; Group 2, control MVs; Group 3, hGC33 scFv‐melittin MVs; Group 4, free nP18 + US; Group 5, nP18@control MVs + US; Group 6, nP18@ hGC33 scFv‐melittin MVs + US. The group containing nP18 was treated with an US wave (5 min, power density 0.8 W/cm^2^, frequency 40 kHz) 24 h post‐intravenous injection. The dosage of P18 was 4 mg/kg. H22‐GPC3 tumors expressed MMP14 highly (Figure S27), which created conditions that encourage for melittin release. Similarly, the size of the tumors and weights of the mice were measured every day for 14 days from the first treatment. The nP18@hGC33 scFv‐melittin MVs + US group inhibited tumor growth significantly compared to any other group (Figures 6b, S28, and S29). Remarkably, the hGC33 scFv‐melittin MVs had an identical impact on tumor growth; this may be due to the cytotoxicity effect of melittin. Body weight remained basically stable (Figure 6c), indicating the safety of the *in vivo* application of this system. We also conducted H&E staining of dissected tumor tissues after mice were sacrificed to confirm the antitumor effect (Figure S30). The nP18@hGC33 scFv‐melittin MVs + US group resulted in more cell necrosis than other groups. The immunohistochemistry staining of hypoxia‐inducible factor (HIF)‐1α was evaluated (Figure 6d). A prominent decrease in the HIF‐1α‐positive signal was observed for the US‐treated nP18@hGC33 scFv‐melittin MVs group relative to the other treatment groups. Hypoxia relief and enhanced drug penetration improved the effect of SDT. Typical blood biochemistry indices were evaluated (Figure S31) and no obvious change was found. These results demonstrated that VAM combined with SDT effectively inhibited tumor growth.

To dissect the nP18@hGC33 scFv‐melittin MVs elicited by antitumor immune response, tumor tissues of all groups were collected to monitor the cellular immune response in the TME. The proportion of CD4^+^ T and CD8^+^ T cells in the TME of nP18@hGC33 scFv‐melittin MVs + US group was significantly up‐regulated, especially for CD8^+^ T cells (Figure 6e). The peripheral blood monocytes (PBMCs), spleens, and lymph nodes from each group were collected at the monitoring terminal. We analyzed the features of lymphocytes in PBMCs, spleens, and lymph nodes. For the nP18@hGC33 scFv‐melittin MVs + US group, the proportion of CD3^+^CD4^+^ T cells and CD3^+^CD8^+^ T cells in lymph nodes was upregulated (Figures 6f,g, S32, and S33). A greater proportion of CD3^+^CD4^+^ T cells in PBMCs was found in the hGC33 scFv‐melittin MVs and nP18@hGC33 scFv‐melittin MVs groups (Figures 6h and S34); For CD3^+^CD8^+^ T cells in peripheral blood monocytes (PBMCs), the hGC33 scFv‐melittin MVs group was slightly higher than nP18@control MVs, and no obvious difference was observed among the other groups (Figures 6i and S35). The number of CD3^+^CD4^+^ T cells in the spleen increased in the nP18@hGC33 scFv‐melittin MVs + US groups (Figures 6j and S36). For CD3^+^CD8^+^ T cells in spleen, both the hGC33 scFv‐melittin MVs and nP18@hGC33 scFv‐melittin MVs + US groups increased (Figures 6k and S37). What's more, the number of CD80^+^CD86^+^ DCs was significantly higher in nP18@hGC33 scFv‐melittin MVs + US group (Figures 6l and S38). The number of CD11b^+^F4/80^+^ macrophages in the spleen (Figures 6m and S39) increased in both the hGC33 scFv‐melittin MVs and nP18@hGC33 scFv‐melittin MVs + US groups. The data revealed that hGC33 scFv‐melittin MVs contributed to the immune response activation. When combined with SDT, more DCs and T cells were activated, which enhanced tumor clearance. These results confirmed that nP18@hGC33 scFv‐melittin MVs acted as an immunoregulator activating the immunosuppressive TME.

## 3 DISCUSSION

Although various modalities have been developed within cancer therapy, including chemotherapy,^42^ targeted therapy,^43^ immunotherapy,^44^ and so forth, countless roadblocks still prevent patients from achieving life‐altering, durable survival. Well‐designed systems are urgently needed to advance the field. Ideally, the optimal regimen would maximize synergistic effects while minimizing overlapping toxicities. Targeted therapy combined with conventional chemotherapy or emerging therapies such as SDT, especially the induction of antitumor immunity, sheds light on the design of efficient therapeutic strategies. In this work, we designed a VAM platform for targeted cancer combination therapy. VAM represents a multifunctional synergistic modality for increased therapeutic efficacy. When combining hGC33 scFv‐melittin MVs with chemotherapeutic DOX, the synergistic effect was reflected in the reduced dosage of DOX while still avoiding overlapping systemic toxicity. Furthermore, when combining hGC33 scFv‐melittin MVs with SDT, enhanced accumulation and penetration of drugs in tumors were found owing to incorporate mechanisms: membrane pore formation and sonoporation. Remarkably, nP18@hGC33 scFv‐melittin MVs induced a robust immune response, which exemplifies the tremendous potential of combining VAM with immunotherapy. Therefore, this VAM represents a proof‐of‐concept combinatorial tumor therapeutics.

In this study, melittin was chosen as the cytotoxic payload for its potential applicability as a cancer therapeutic agent that is less likely to develop resistance compared with traditional chemotherapeutics. Antibody‐drug conjugates (ADCs) for treating solid tumor have gradually become a research hotpot since ADCs were first approved to treat hematologic malignancies therapy.^45^ Acquiring adequate penetration depth in solid tumors is the key to achieving a therapeutic effect. There is no doubt that melittin is an ideal chaperone in terms of promoting drug penetration. Through genetically engineering, the heterogeneity of drug‐antibody ratio existing in chemical modification can be minimized. Due to the MMP14 responsive linker between hGC33 scFv and melittin, melittin could be released specifically at MMP14‐expressing tumor sites.^46^, ^47^, ^48^ The MVs displaying target protein could achieve multimodal therapeutics using the membrane‐displaying technique developed by our group.^19^, ^40^ MVs originating from natural cell membranes have been exploited as an attractive drug delivery system toward clinical applications.^49^, ^50^ In this work, chemotherapeutic DOX or nanosonosensitizer nP18 was loaded into this natural carrier, and its synergistic antitumor effects were verified in different animal models. For DOX@hGC33 scFv‐melittin MVs, no serious adverse effects (e.g., cardiotoxicity) were found after intravenous administration in HepG2‐bearing Balb/c nude mice. For nP18@hGC33 scFv‐melittin MVs, fluorescence imaging‐guided SDT activated the immune response in H22‐GPC3‐bearing Balb/c immune‐competent mice.

In summary, here we presented a VAM platform for multimodal synergetic cancer therapy. Genetically engineered hGC33 scFv‐melittin MVs‐derived drugs showed coordinated treatment effects, which warrant further exploration for clinical application. Immunotherapy is now a pillar of cancer treatment,^51^, ^52^ and novel treatment combinations have emerged as attractive immunotherapeutic approaches. VAM combined with SDT potentiated the effects of activating immune cells and regulating the TME through multiple mechanisms. The VAM platform we designed provides a new avenue for synergistic cancer combination immunotherapies with transformational clinical potential.

## 4 METHODS AND MATERIALS

### 4.1 Construction of stable cell lines

The 5 × 10^4^ BHK‐21 cells (Baby hamster kidney cell line, Laboratory storage) were inoculated in a 24‐well plate, and infected by lentiviruses containing target gene sequence until cells fusion rate was 60%–70%, 5 μg/ml polybrene (GenePharma, Shanghai, China) was added while infection for introducing lentiviruses into mammalian cells. Replacing fresh medium 24 h post‐infection, and monitoring the EGFP (Enhanced green fluorescence protein) expression by fluorescence microscope (Nikon) 72–96 h post‐infection. Continuous cell passage was carried out, and screening stable cell lines by adding 4 μg/ml puromycin (Solarbio, Beijing, China). The gene sequences of hGC33 scFv were obtained from IMGT (https://www.imgt.org/). Cells were cultured in DMEM (Dulbecco's Modified Eagle's Medium) supplemented with 10% fetal bovine serum and penicillin/streptomycin at 37°C incubator with a humidified 5% CO_2_ atmosphere.

### 4.2 Preparation of MVs

While the cell fusion rate reached 90% in 10‐cm cell culture dish, removing the supernatant from the medium, washing three times with cold PBS (10 mM), and collecting cells to centrifuge tubes by cell scraper (Beyotime Technology, China). Resuspending cells with PBS (Phosphate buffered saline) containing 1 mM PMSF (Phenylmethylsulfonyl fluoride, Beyotime technology, China), and the cells were treated by ultrasonic wave (three times, 10 s each) under an ice bath. Then MVs were obtained by multistep density gradient ultracentrifugation.^19^ In brief, the cell resuspension solution was centrifuged at 3000 rpm/10 min/4 °C; discarding the pellet, and collected supernatant was centrifuged at 5000 rpm/10 min/4 °C; discarding the pellet, and finally collected supernatant was centrifuged at 15,000 rpm/60 min/4 °C. The isolated MVs could be sheared to the desirable size with various size membrane filters (Whatman). For drug encapsulation, mixing the drug and MVs, and then the mixture was extruded by a mini‐extruder (Avanti, USA), finally, the drug‐loaded MVs were obtained by centrifuge. For encapsulation efficiency, it was determined by the calibration curve of DOX absorbance at 514 nm. The loading capacity of DOX is 74.52% ± 4.13% (For encapsulating 40 μg DOX into 100 μg MVs). The MVs were preserved at −80°C until usage. The MVs were quantified via BCA Protein Assay Kit (Pierce).

### 4.3 Morphology, size, and potential

The TEM (Tecnai G2 Spirit BioTwin) at 80 kV acceleration voltage was used to observe the morphology of the nanoparticles. The hydraulic diameter and zeta potential of nanoparticles were obtained by a DLS (Malvern).

### 4.4 Western blotting analysis

Preparing gels containing 10% acrylamide for target protein detection. Adding loading buffer to the samples and then treated by heat at 95°C for 10 min to denature proteins. For protein extraction of cells, protein was obtained by Protein Extraction Kit (Sangon, Shanghai, China) according to the manufacturer's procedure; For protein extraction of tissues, 100 mg tissue were weighed and homogenized, then treated by ultrasonic wave under ice bath, and the following procedure was the same as the protein extraction of cells. Loading the samples and marker to the wells of SDS‐PAGE (Sodium dodecyl sulfate‐polyacrylamide gel electrophoresis) gel, running the samples at a constant volt of 80 V for 30 min, and following 120 V for 90 min. Choosing PVDF (Polyvinylidene fluoride, Whatman) for blotting membrane and electroblotting was at a constant volt 100 V for 60 min. Blocking the membrane using 5% skim milk (Beyotime Technology, China) to prevent nonspecific binding of antibodies. Transferring the membrane to the primary antibody solution (1:1000–1:2000) and shaking overnight at 4 °C, washing the membrane three times for 5 min with PBS‐tween20 (0.5%); Transferring the membrane to the secondary antibody solution (1:5000) and shaking at room temperature (RT) for 1 h, washing the membrane three times for 5 min with PBS‐tween20. Finally, placing the membrane inside the chemiluminescence detector (Bio‐Rad ChemiDoc XRS System) and take a picture using a CCD camera. For hGC33 scFv detection, the primary antibody was human GPC3 protein (His tag) (Sino biological, China), and the secondary antibody was HRP‐conjugated anti‐His monoclonal antibody (Sangon, Shanghai, China). Na^+^/K^+^‐ATPase α1 was selected as a reference (Abp51894, Abbkine). For MMP14 detection, the primary antibody was anti‐MMP14 rabbit monoclonal antibody (ab51074, Abcam), and the secondary antibody was HRP‐conjugated goat antirabbit monoclonal antibody (Sangon, Shanghai, China). GAPDH (Glyceraldehyde‐3‐phosphate dehydrogenase) was selected as reference (AG019, Beyotime technology).

### 4.5 Flow cytometry analysis

Rinsing the cells with PBS, and resuspending cells with flow cytometry staining buffer (Lonza technology, China), after blocking, adding appropriate amount of antibody to each compensation tube and staining at RT for 30 min in the dark. Removing supernatant carefully and resuspending the cells with 200–400 μl of FACS buffer. For flag tag detection inside the membrane, a procedure disrupting the membrane was needed, then the Alexa Fluor 647‐conjugated anti‐DDDDK tag antibody (ab245893, Abcam) was used. For hGC33 scFv detection outside the membrane, the primary antibody was human GPC3 protein (His tag) (Sino biological, China), and the secondary antibody was APC (Allophycocyanin)‐conjugated anti‐His tag antibody (J095G46, Biolegend). For apoptosis assay, Annexin V‐FITC (Fluorescein isothiocyanate)/PI (Propidium iodide) was purchased from Sangon biotechnology, China. For surface markers analysis of lymphocytes: PE‐conjugated antimouse CD4, FITC‐conjugated antimouse CD8α, FITC‐conjugated antimouse CD80, APC‐conjugated antimouse CD86, APC‐conjugated antimouse CD3, APC‐conjugated antimouse CD11b, FITC‐conjugated antimouse F4/80, PE‐conjugated antimouse CD16/32, and PE‐conjugated antimouse CD11c were purchased from Biolegend, USA. The instrument in this article was Beckman Cytoflex, and data were analyzed by FlowJo version 10 software.

### 4.6 Immunofluorescence

The 2 × 10^5^ cells were inoculated in the confocal dish (20 mm, Nest), and then cultured in the incubator at 37°C overnight. Removing the medium and washing three times with cold PBS. 4% paraformaldehyde (Biosharp, China) was used to fix the cells at RT for 15 min, washing three times with PBS; Using PBS‐triton X‐100 to make cells permeable (20 min at RT), washing three times with PBST (Phosphate buffered solution), and blocking cells with 5% BSA (ServiceBio, China) at RT for 1 h; Washing three times with PBST, adding appropriate amount of primary antibody to the dish and co‐incubation at RT for 1 h; Washing three times with PBST, adding secondary antibody to the dish and co‐incubation at RT for 1 h; Finally, Washing three times with PBST, and using DAPI (Lablead, China) to stain the nucleus. Laser scanning confocal microscopy (LSCM) was used to analyze the fluorescence (Olympus FV1200).

### 4.7 Cell‐based ELISA

The 2 × 10^4^ HepG2 cells were inoculated in a 96‐well plate, washing three times with cold PBS after 24 h culturing. The cells were fixed with precooled 0.05% glutaraldehyde (Aladdin, China) for 15 min. Washing three times with PBS, blocking cells with 1% BSA at 4 °C overnight. Washing three times with PBS, adding samples to be tested to the wells (Each group had three parallels), and incubating at 37°C for 2 h. Washing three times with PBS‐tween 20, then adding antiflag tag monoclonal antibody (1:1500, CST), incubating at 37 °C for 1 h. Washing three times with PBS‐tween 20, adding HRP‐conjugated goat antimouse IgG (Immumoglobulin G, 1:2000, Sangon, Shanghai, China), incubating at 37°C for 1 h. Washing three times with PBS‐tween 20, adding substrate, and incubating at 37°C for 15 min in the dark, using microplate reader (Multiscan Go, Thermo Scientific) to detect the absorbance at 405 nm after the reaction stopped.

### 4.8 *In vitro* cell uptake

The 2 × 10^5^ HepG2 or L02 cells were inoculated in the confocal dish and cultured overnight, replacing fresh medium, and adding corresponding samples to the dish and co‐incubated for 4–6 h. Removing the medium and washing three times with cold PBS. Using 4% paraformaldehyde to fix the cells and staining with DAPI for 3 min at RT. If the cytoskeleton was needed to be indicated, phalloidine (Servicebio, Wuhan, China) was applied for staining at RT for 20 min and washed three times with PBS. For targeting ability verification, MVs were labeled by DiI (MedChemExpress). The concentration of DOX was 2 μg/ml and p18 was 10 μg/ml.

### 4.9 3D tumor spheroids assay

Weighing agarose and dissolved with deionized (DI) water to 10 mg/ml, heating by microwave, and adding soluble agarose to a 96‐well plate as soon as possible. Setting the plate under UV light for 30 min, adding 1 × 10^4^ HepG2 cells to each well, and culturing in the incubator for 5–6 days until sphere forming. For cell uptake, transferring the spheroid to the confocal dish, adding samples to be tested and co‐incubated for 4–6 h, washing three times with PBS. Finally, the tumor spheroids were observed qualitatively and quantitatively by the LSCM (Zeiss LSM 880 + Airyscan). For growth inhibition assay, the morphology of spheroids was observed by light microscope (Nikon), the surface area of spheroids was measured by image J software.

### 4.10 Penetration study in *ex vivo* tumor tissues

H22‐GPC3 tumor tissues were dissected from Balb/c mice, and washed with PBS to remove blood. Hundred milligram tumor tissues were weighed and treated by ultrasonic wave (0.8 W/cm^2^, Chattanooga 2776, USA) for 5 min. Free nP18, nP18@control MVs, and nP18@hGC33 scFv‐melittin MVs (based on the concentration of P18: 10 μg/ml) were co‐incubated with tumor tissues at 37°C overnight. Washing three times with PBS. Freezing the tumor tissues embedded with optimum cutting temperature (OCT) compound (SAKURA) immediately at −80°C, and making frozen tissue slices with a frozen slicer blade (SLEE MEV, Germany), each slice set at 10 μm. Slices were observed by LSCM (Excitation wavelength: 640 nm).

### 4.11 Cell cytotoxicity assay

The 1 × 10^4^ HepG2 cells were inoculated in a 96‐well plate, and cultured overnight, replacing fresh medium, and adding corresponding samples to wells and co‐incubated for 24 h. Washing once with PBS, and cells were measured by Cell Counting Kit‐8 assay (Lablead, Beijing, China). In brief, 100 μl medium and 10 μl kit were added to each well, setting the plate at 37 °C for 1–2 h and measured at 450 nm by microplate reader (Multiscan Go, Thermo Scientific). The cell viability of each sample was calculated based on the following formula:

$$\text{Cell viability}\;\left(\%\right)=\frac{{\mathrm{OD}}_{\text{sample}}-{\mathrm{OD}}_{\text{background}}}{{\mathrm{OD}}_{\text{control}}-{\mathrm{OD}}_{\text{background}}}\times 100\%$$

### 4.12 Crystal violet staining

The 5 × 10^4^ HepG2 cells were inoculated in a 24‐well plate and cultured overnight, removing the medium and washing three times with cold PBS. Adding free DOX (BBI life sciences, China), DOX@control MVs, DOX@hGC33 scFv‐melittin MVs (the concentration of DOX was 2 μg/ml) to the wells, and incubated for 24 h at 37°C. Removing the medium and washing twice with PBS, fixing cells with 1% methanol (Sinopharma Chemical, China) for 30 s. Each well was added with 200 μl crystal violet dye (Aladdin, China) at RT for 20 min, then washed with distilled water until the supernatant was clear.

### 4.13 Hemolytic test

Collecting blood from orbital of Balb/c mice with an anticoagulant tube, and centrifuged at 3000 rpm for 20 min, removing upper plasma carefully. Rinsing lower red blood cells with saline for three times until the supernatant was clear. Preparing 2% red blood cells for hemolytic test. Adding the above red blood cells to a 96‐well plate, samples to be tested were added to wells and co‐incubated at 37°C for 2 h. The plate was centrifuged at 1000 rpm for 15 min by centrifuge (Eppendorf), transferring the supernatant to another 96‐well plate, and detected at 414 nm by microplate reader (Multiscan Go, Thermo Scientific).

### 4.14 *In vivo* blood circulation

The blood retention time of P18 was studied by Balb/c mice. Free nP18 and nP18@MVs (based on the concentration of P18: 4 mg/kg) were intravenously into the mice, respectively. Collecting orbital blood (100 μl) with anticoagulant tube at 0, 1, 3, 6, 9, 12, 24, 36, and 48 h postinjection. Transferring the blood to a black 96‐well plate and detecting the fluorescence by IVIS (Caliper IVIS Lumina II).

### 4.15 *In vivo* fluorescence imaging

The targeting capacity of nP18@hGC33 scFv‐melittin MVs was investigated by Balb/c mice bearing H22‐GPC3 subcutaneous tumor. Free nP18, nP18@control MVs, nP18@hGC33 scFv‐melittin MVs (based on the concentration of P18: 4 mg/kg) were intravenously into H22‐GPC3 bearing mice, respectively. The fluorescence imaging was performed by IVIS (Caliper IVIS Lumina II) at 12, 24, and 36 h postinjection. At the monitoring terminal, mice were sacrificed, tumors and major organs were excised for *ex vivo* imaging.

### 4.16 Immunohistochemistry staining

The harvested tumor tissues were washed with saline and immediately fixed in 4% paraformaldehyde, then processed for paraffin embedding. After antigen retrieval, sections were incubated with anti‐HIF1α antibody (1:100 dilutions, Boster Biological) overnight at 4°C. Then sections were incubated with secondary antibody (1:200 dilutions) for 2 h at RT and counterstained for 30 s. The images were detected using an optical microscope (Nikon Ti‐U).

### 4.17 Separation of lymphocyte populations

For spleen lymphocytes collection, spleen was harvested under aseptic conditions, and cut into small pieces with ophthalmic scissors. Placing the above spleen on a filter (70 μm, JingAn Biological, China) and using a syringe piston to grind the spleen. Then Cell suspension was collected, single cell suspension of spleen was prepared according to manufacturer's protocol (Solarbio, China). Briefly, single cell suspension of spleen was added to liquid surface of the 3 ml separation reagent carefully in a 15 ml centrifuge tube. After centrifugation (1000 g, 30 min, RT), the middle milky lymphocyte layer was transferred to another centrifuge tube, washed three times, and lymphocyte populations were obtained.

For peripheral blood lymphocytes collection, anticoagulant blood was added to the liquid surface of the 3 ml Ficoll reagent (Yuanye Bio, China) carefully in a 15 ml centrifuge tube. After centrifugation (1000 g, 30 min, RT), transferring lymphocyte layer to another centrifuge tube and removing erythrocytes with ACK lysis buffer (Beyotime Technology, China). Finally, washing three times, and lymphocyte populations were obtained.

### 4.18 *In vivo* antitumor efficacy

Specific pathogen‐free (SPF) 4–6 weeks female Balb/c nude mice were purchased from Shanghai SLAC Laboratory Animal Co, Ltd. SPF 4–6 weeks female Balb/c mice were purchased from Beijing Vital River Laboratory Animal Technology Co. Ltd. All animal studies were conducted under the Institutional Animal Care and Use Committee (IACUC) of Xiamen University.

For HepG2 bearing Balb/c nude mice model, 5 × 10^6^ HepG2 cells were subcutaneously injected into the right flank of mice. The mice were randomly divided into six groups while the tumor volume reached ~80 mm^3^. Hundred microliter prepared drugs were intravenously administrated to the mice every day for three times (based on the concentration of DOX of 1 mg/kg). For H22‐GPC3 bearing Balb/c immune‐competent mice model, 5 × 10^6^ H22‐GPC3 cells were subcutaneously injected into the right flank of mice (mice were molted beforehand). Similarly, the mice were randomly divided into six groups while the tumor volume reached ~80 mm^3^. 200 μl prepared drugs were intravenously administrated to the mice every 2 days for three times (based on the concentration of P18 of 4 mg/kg), and the tumors were subjected to ultrasonic wave (0.8 W/cm^2^, 5 min) 24 h postinjection. The tumor volume and body weight were monitored every day, and the tumor volume was calculated according to the formula: volume (*V*) = (length × width^2^)/2.

During the treatment, peripheral blood was collected in each group for the evaluation of biochemical indexes. In order to verify the biological safety, indicators including ALT, AST, CREA, and CK‐MB were measured.

At the terminal of monitoring, major organs including heart, liver, spleen, lung, kidney, lymph nodes, and tumors were harvested for tissue slices or lymphocytes assessment.

### 4.19 Statistical analysis

Statistical analyses were performed using GraphPad Prism 8.0 software. Comparisons among multiple groups were performed using a one‐way ANOVA. Comparisons between two groups were performed using unpaired Student's *t*‐tests. The *p* values of <0.05 were considered significant. *p < 0.05, **p < 0.01, ***p < 0.001, and ****p < 0.0001. ns, not significant.

## AUTHOR CONTRIBUTIONS

Jianzhong Zhang, Xue Liu, and Gang Liu conceived and designed the experiments. Jianzhong Zhang, Xue Liu, Yutian Xia, Shuyu Xu, Xuan Liu, Haiqing Xiao, Xiaoyong Wang, and Chao Liu performed the experiments. Jianzhong Zhang, Xue Liu, Chao Liu, and Gang Liu analyzed the results. Jianzhong Zhang, Xue Liu, and Gang Liu wrote the article. Gang Liu supervised the entire project.

## FUNDING INFORMATION

This work was supported by the Major State Basic Research Development Program of China (2017YFA0205201), the National Natural Science Foundation of China (NSFC) (81925019, U1705281, U22A20333, and 82272144), the Fundamental Research Funds for the Central Universities (20720190088, 20720200019, and 2020Y4003), the Program for New Century Excellent Talents in University, China (NCET‐13‐0502), and China Postdoctoral Science Foundation (2021T140399).

## CONFLICT OF INTEREST

The authors declare no conflict of interest.

## Supporting information


**Data S1:** Supporting information

## Data Availability

The main data supporting the results in this study are available within the paper and its supporting information. Additional data related to this work are available for research purposes from the corresponding author on reasonable request.
