# Supplementary material for: Genetically engineered nano‐melittin vesicles for multimodal synergetic cancer therapy
Source: Bioeng Transl Med. 2023 Jan 4;8(6):e10482. doi: 10.1002/btm2.10482 (PMC10658496; doi:10.1002/btm2.10482)
Supplement: Supplementary file 1 — Data S1: Supporting information [file BTM2-8-e10482-s001.docx]

**Supplementary Information**

**Genetically engineered nano-melittin vesicles for multi-modal synergetic cancer therapy**

Jianzhong Zhang^1#^, Xue Liu^1,2#^, Yutian Xia^1^, Shuyu Xu^1^, Xuan Liu^1^, Haiqing Xiao^1^, Xiaoyong Wang^1^, Chao Liu^1*^, Gang Liu^1,3*^

^1^State Key Laboratory of Molecular Vaccinology and Molecular Diagnostics and Center for Molecular Imaging and Translational Medicine, School of Public Health, Xiamen University, Xiamen, 361102, China.

^2^State Key Laboratory of Molecular Vaccinology and Molecular Diagnostics, National Institute of Diagnostics and Vaccine Development in Infectious Diseases, School of Public Health, Xiamen University, Xiamen, 361102, China.

^3^State Key Laboratory of Cellular Stress Biology, Innovation Center for Cell Biology, School of Life Sciences, Xiamen University, Xiamen 361102, China.

^*^ Corresponding Author. E-mail: gangliu.cmitm@xmu.edu.cn (G. Liu); liuchao66888@xmu.edu.cn (C. Liu).

^#^ Jianzhong Zhang and Xue Liu contributed equally to this work.


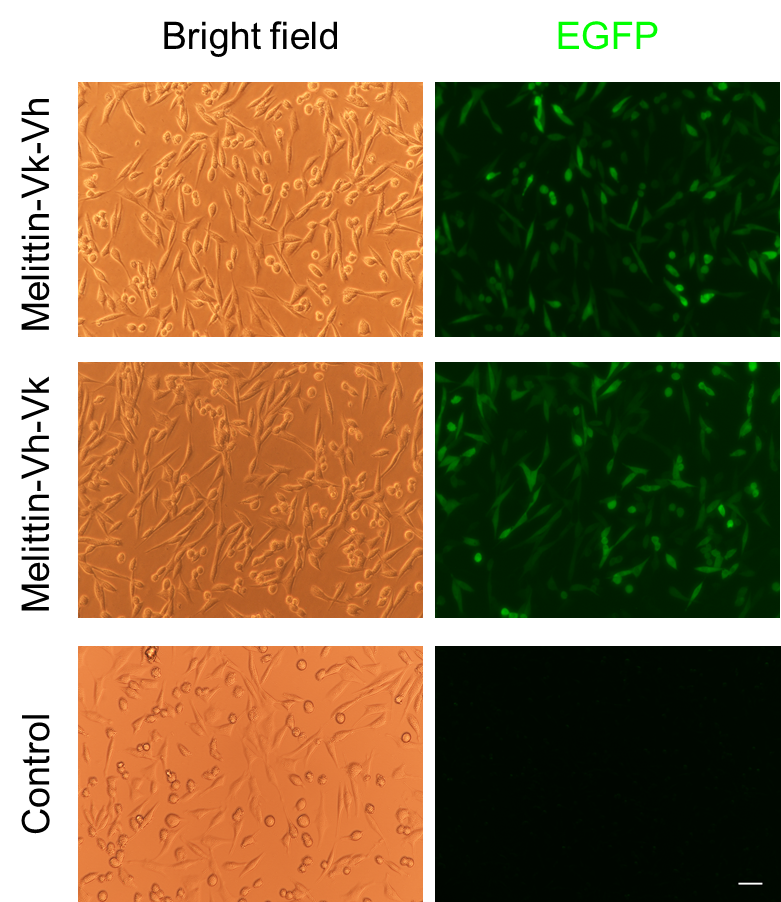


**Figure S1. Target cells (BHK-21) were infected by lenti-viruses carrying EGFP gene, and the positive cells were screened by puromycin. Scale bar: 100 μm.**


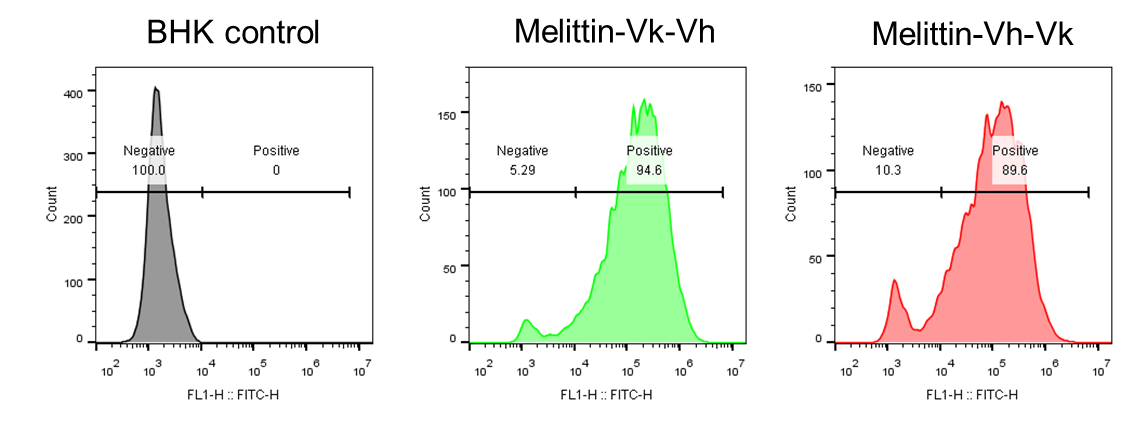


**Figure S2. The EGFP positive cells were detected by flow cytometry.**


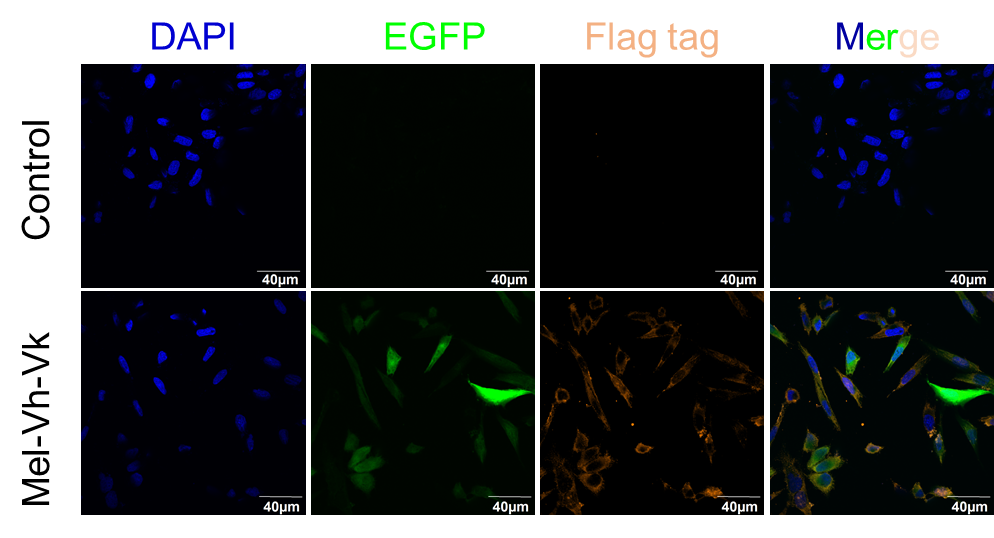


**Figure S3. The detection of recombinant fusion protein by immunofluorescence (IF).** **Blue: DAPI, Green: EGFP, Orange: Flag tag. Scale bar: 40 μm.**


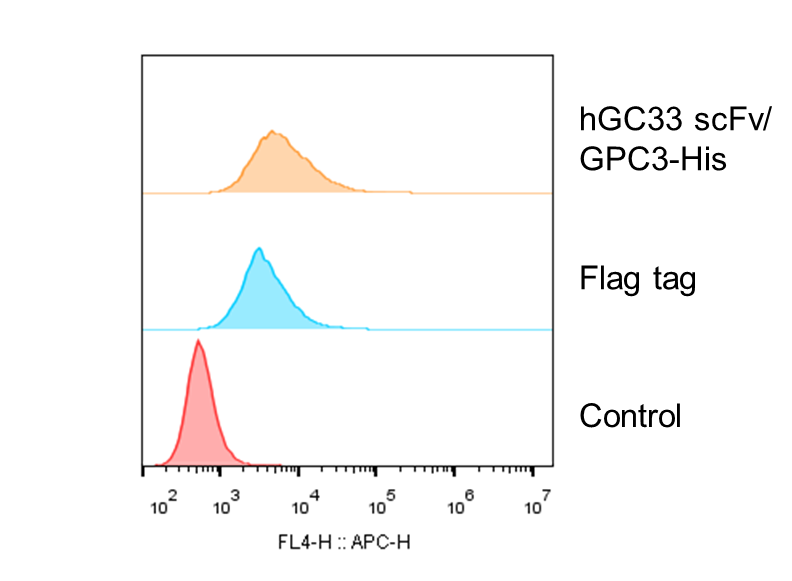


**Figure S4. The detection of recombinant fusion protein by flow cytometry.**


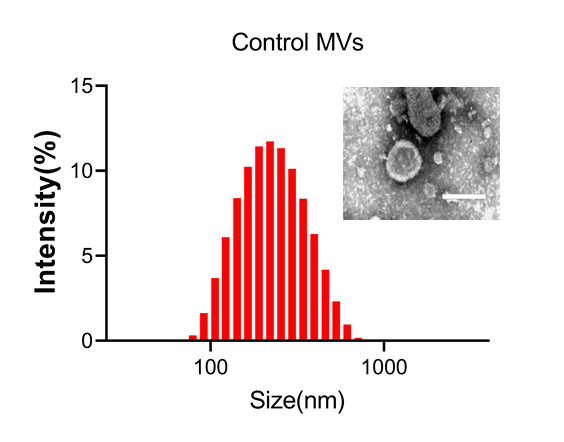


**Figure S5. Morphology images of MVs by transmission electron microscopy (TEM); Size distribution of MVs by dynamic light scattering (DLS). For control MVs, the average diameter is 218.7 nm. Scale bar: 200 nm.**


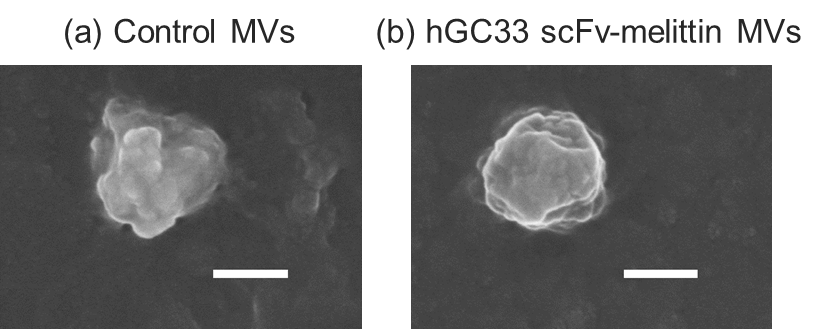


**Figure S6. Morphology images of MVs by scanning electron microscopy (SEM, EHT: 5.00 kV, WD: 9.5 mm, Mag: 80.00 K X; SUPRA55 SAPPHIRE, Germany). Scale bar: 100 nm.**


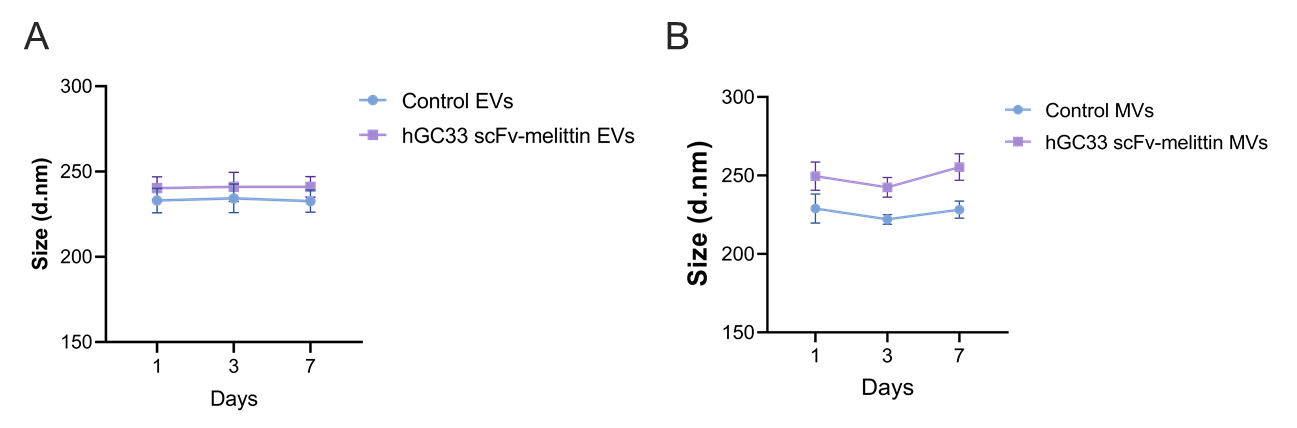


**Figure S7. The size change of MVs at 4 °C in (A) PBS or (B) serum. All the data are presented as means ± SD (n = 3).**


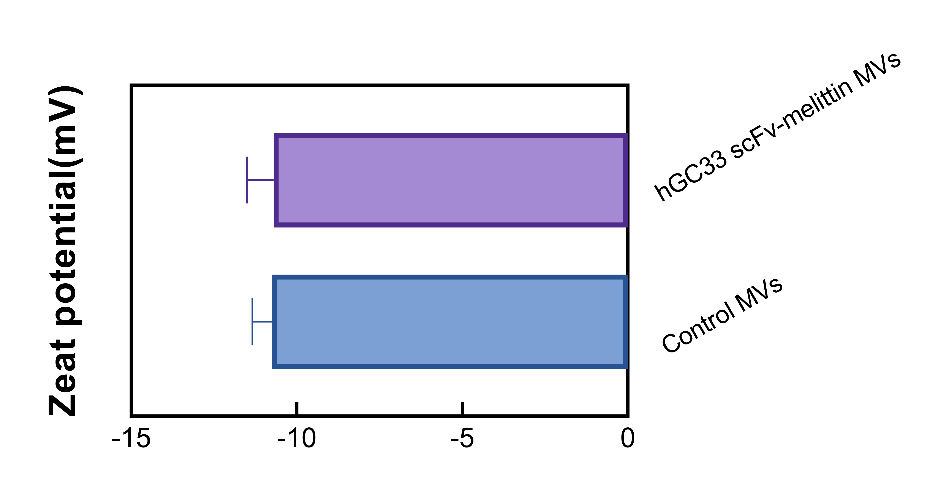


**Figure S8. Zeta potential of MVs. All the data are presented as means ± SD (n = 3).**


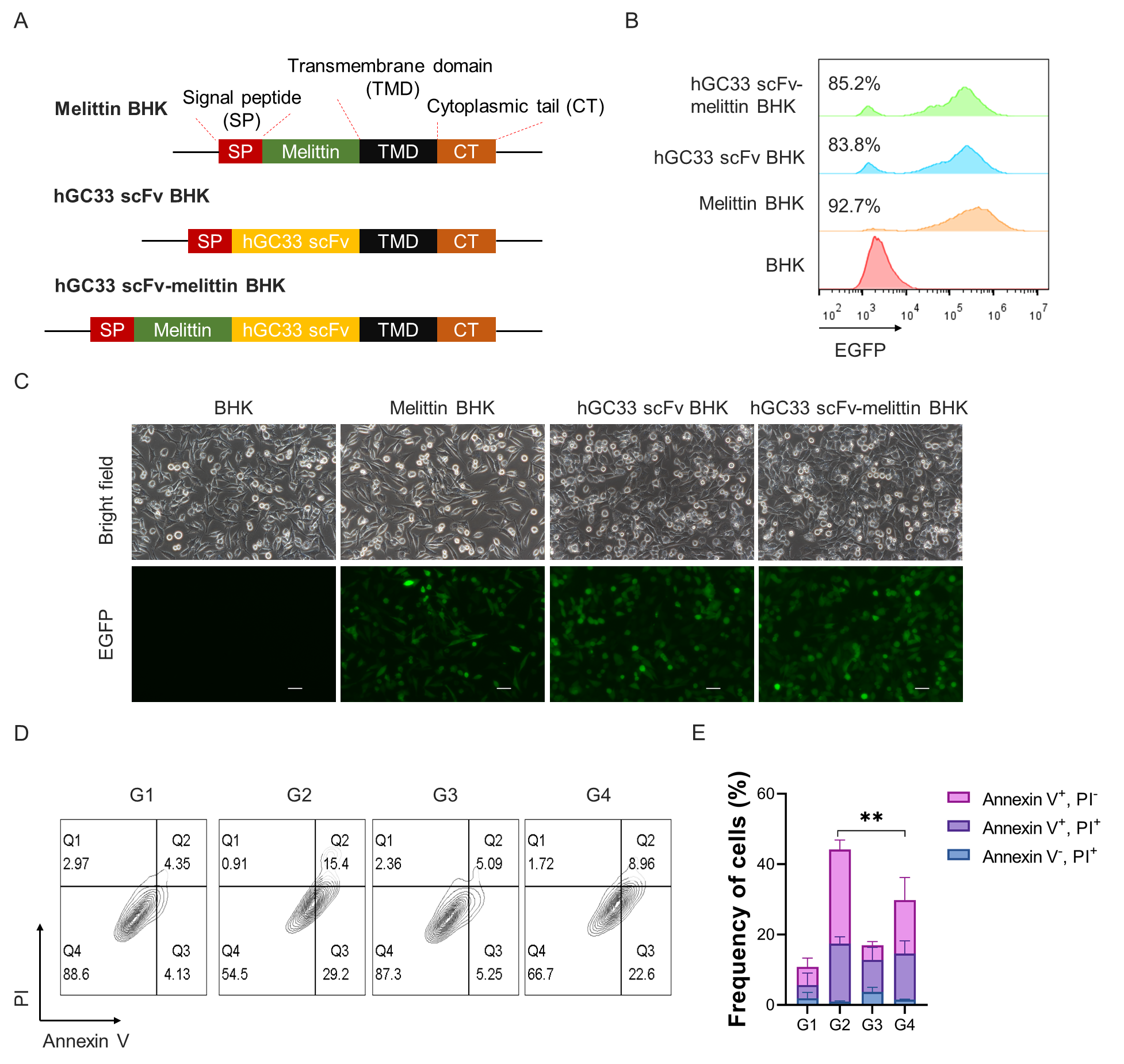


**Figure S9. (A) Schematic diagram of construction strategies of ‘melittin only’, ‘hGC33 scFv only’, and ‘hGC33 scFv-melittin’ recombinant gene fragments. (B) BHK cells were infected by lenti-viruses carrying EGFP gene, and the positive cells were detected by flow cytometry and (C) fluorescence microscope, Scale bar: 100 μm. (D) Apoptosis induced by different MVs (100 μg) in L02 was detected by flow cytometry. (E) Statistical analysis of necrosis (Annexin V^−^, PI^+^), late apoptosis (Annexin V^+^, PI^+^), apoptosis (Annexin V^+^, PI^−^) in different treatment groups. G1, control MVs (none of those MVs); G2, melittin only MVs; G3, hGC33 scFv only MVs; G4, hGC33 scFv-melittin MVs. All the data are presented as means ± SD (n = 3). **p < 0.01.**

**
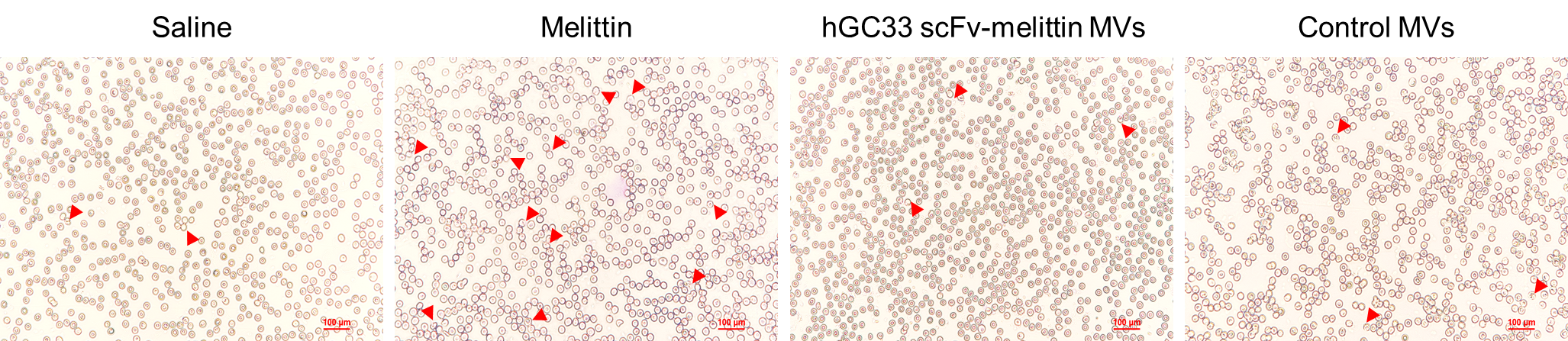
**

**Figure S10. Representative photograph of hemolysis caused by melittin. Scale bar: 100 μm.**

**
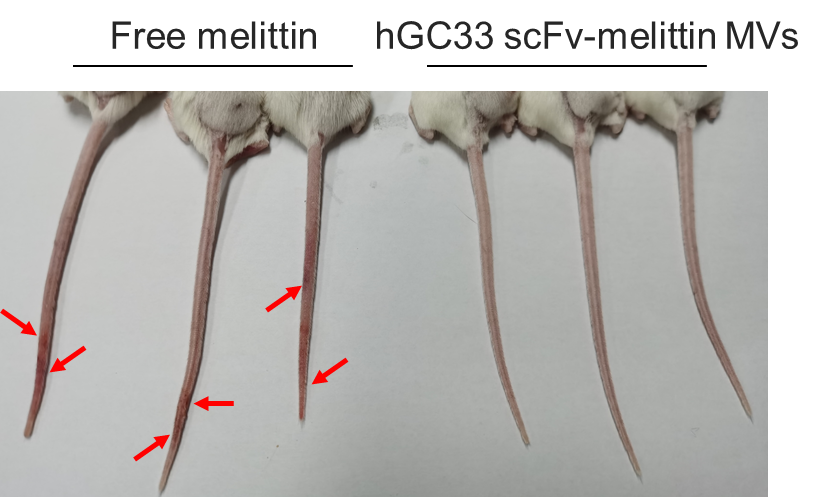
**

**Figure S11. Photos of the tails of different treatment groups at the end point of three injections. Melittin (1 mg/kg), hGC33 scFv-melittin MVs (10 mg/kg).**

**
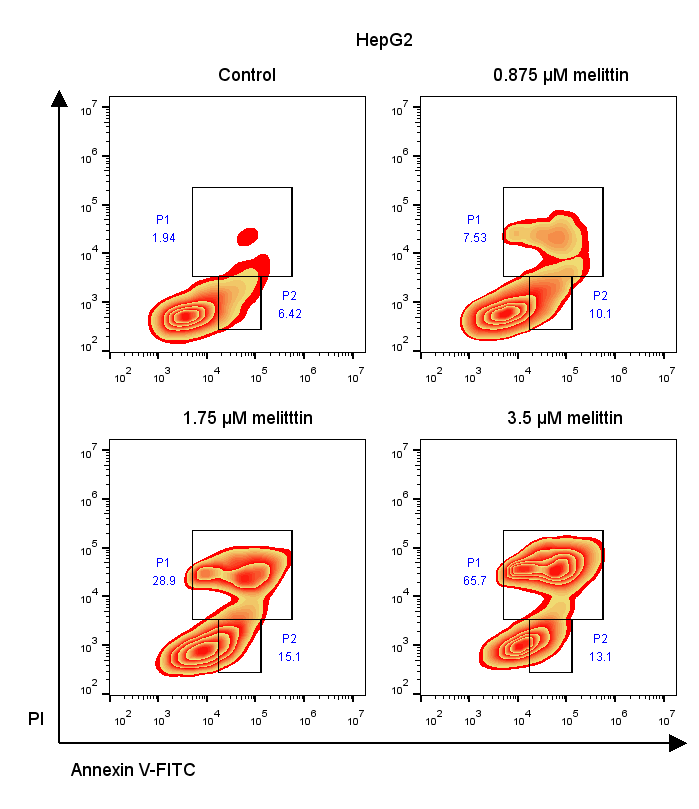
**

**Figure S12. Apoptosis induced by melittin in HepG2 detected by flow cytometry.**

**
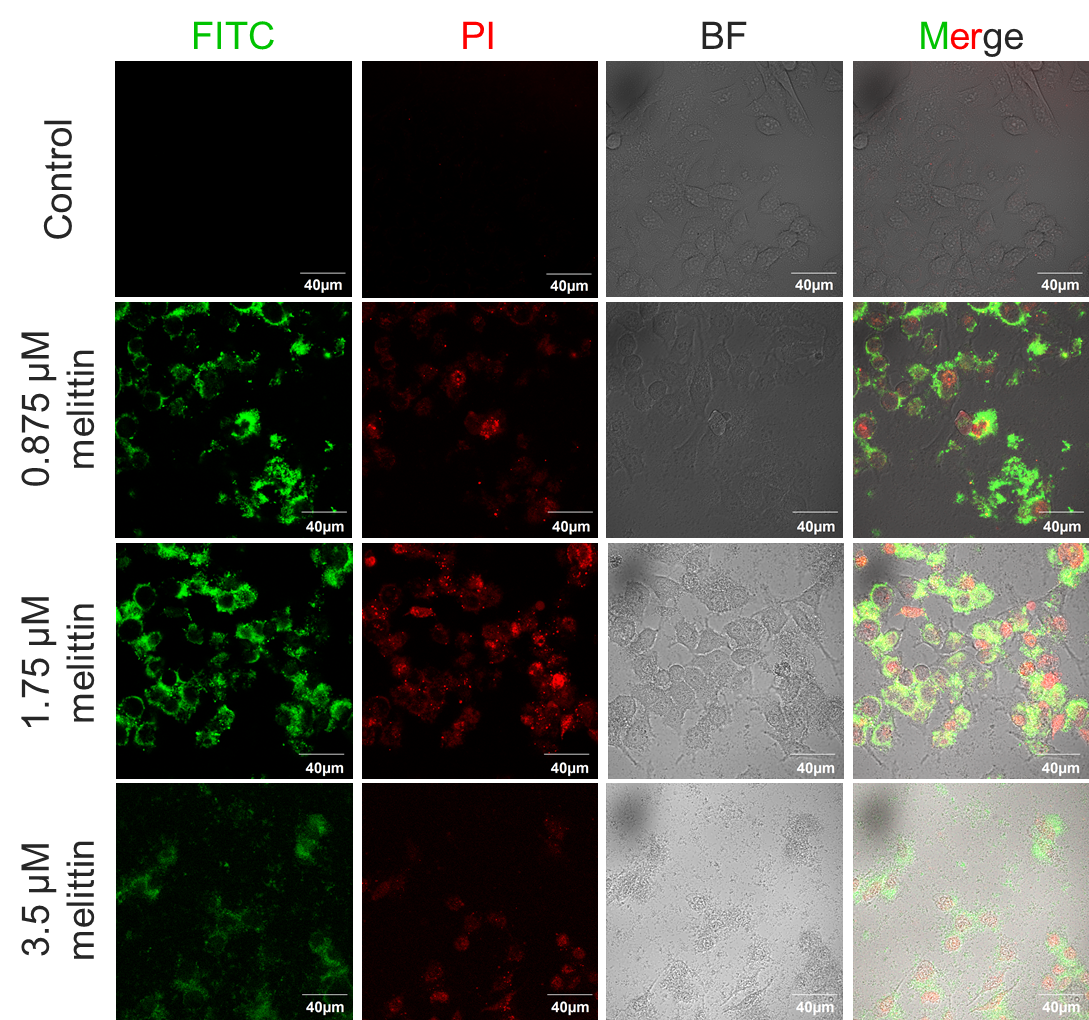
**

**Figure S13. Apoptosis induced by melittin in HepG2 detected by laser scanning confocal microscopy (LSCM). Green: Annexin V-FITC, Red: PI. Scale bar: 40 μm.**


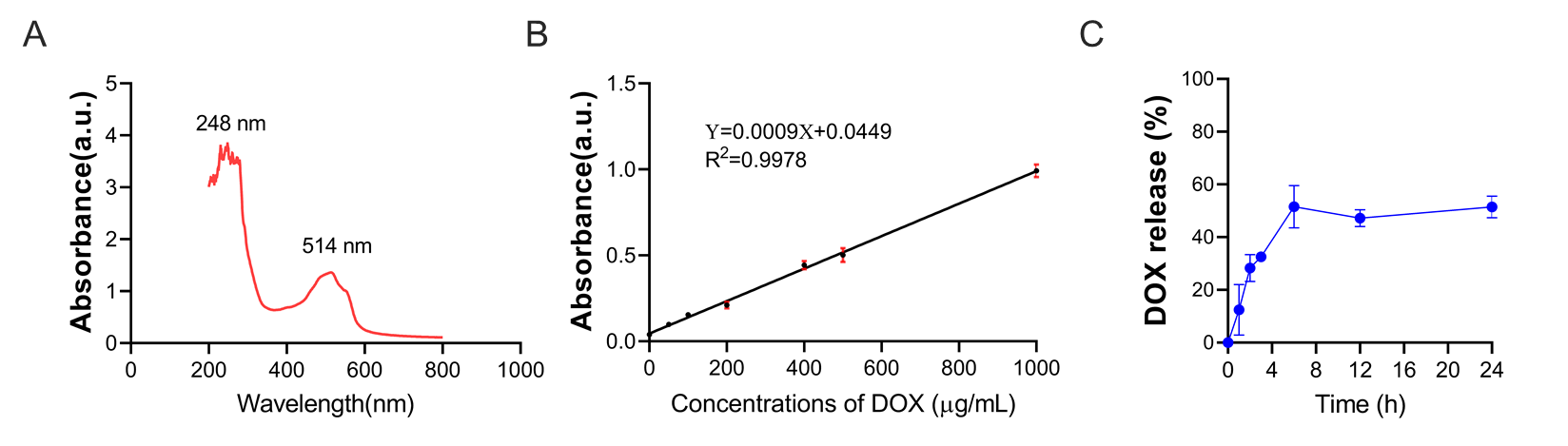


**Figure S14. (A) Absorption spectrum of DOX. (B) Standard curve of DOX in different concentrations. (C) Release profile of DOX from MVs at 37 ℃ in PBS buffer (pH=7.4). All the data are presented as means ± SD (n = 3).**

**
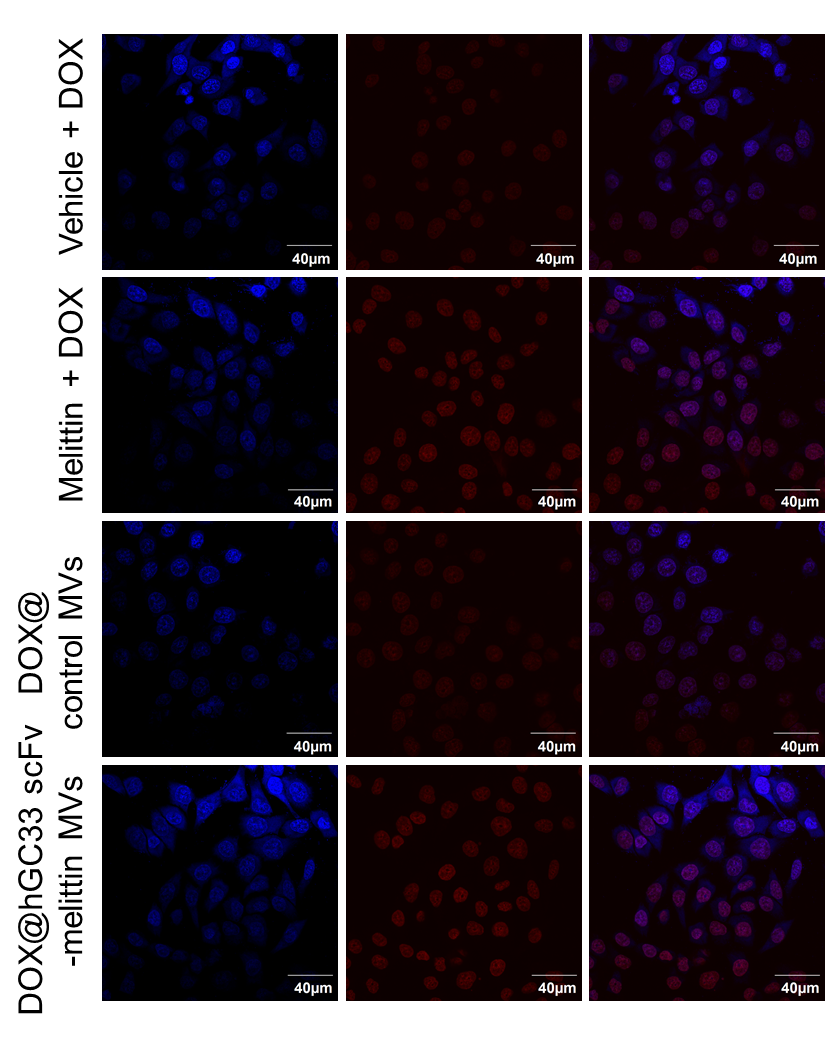
**

**Figure S15. Cell uptaking of DOX in different treatment groups. Blue: DAPI, Red: DOX.** **Scale bar: 40 μm.**

**
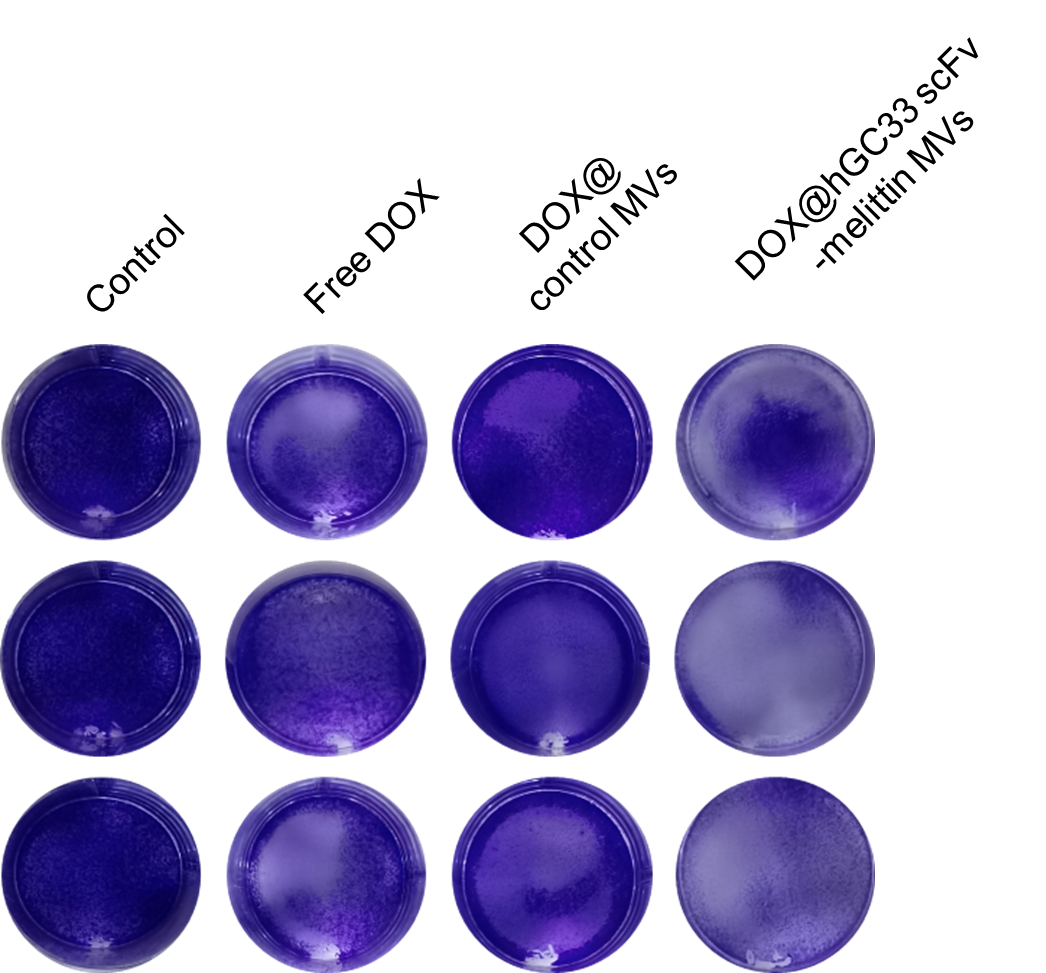
**

**Figure S16. Cell cytotoxicity of DOX in different treatment groups by crystal violet staining.**

**
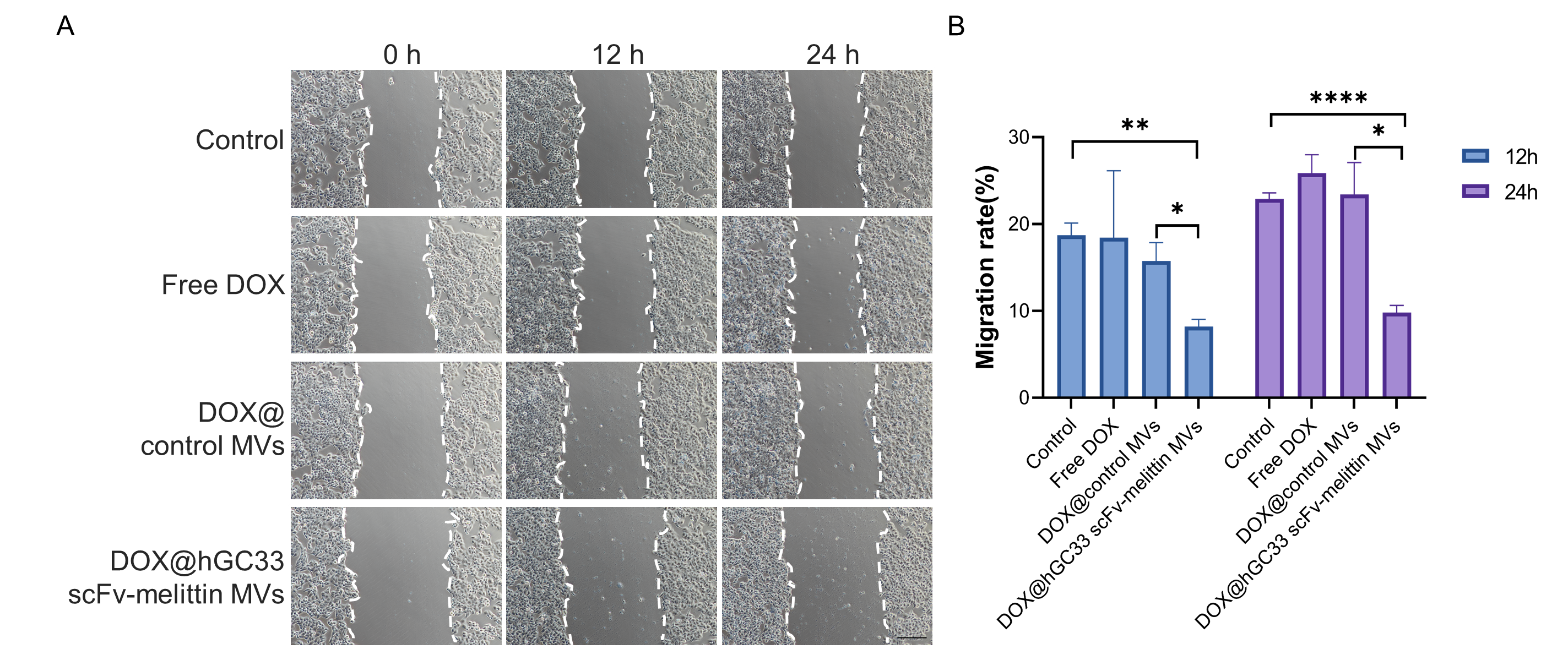
**

**Figure S17. Cell scratch test of HepG2 in each group. (A) Representative images at different time points. (B) Statistical analysis of migration in each group. All the data are presented as means ± SD (n = 3). **p* < 0.05, ***p* < 0.01, *****p* < 0.0001. Scale bar: 200 μm.**

**
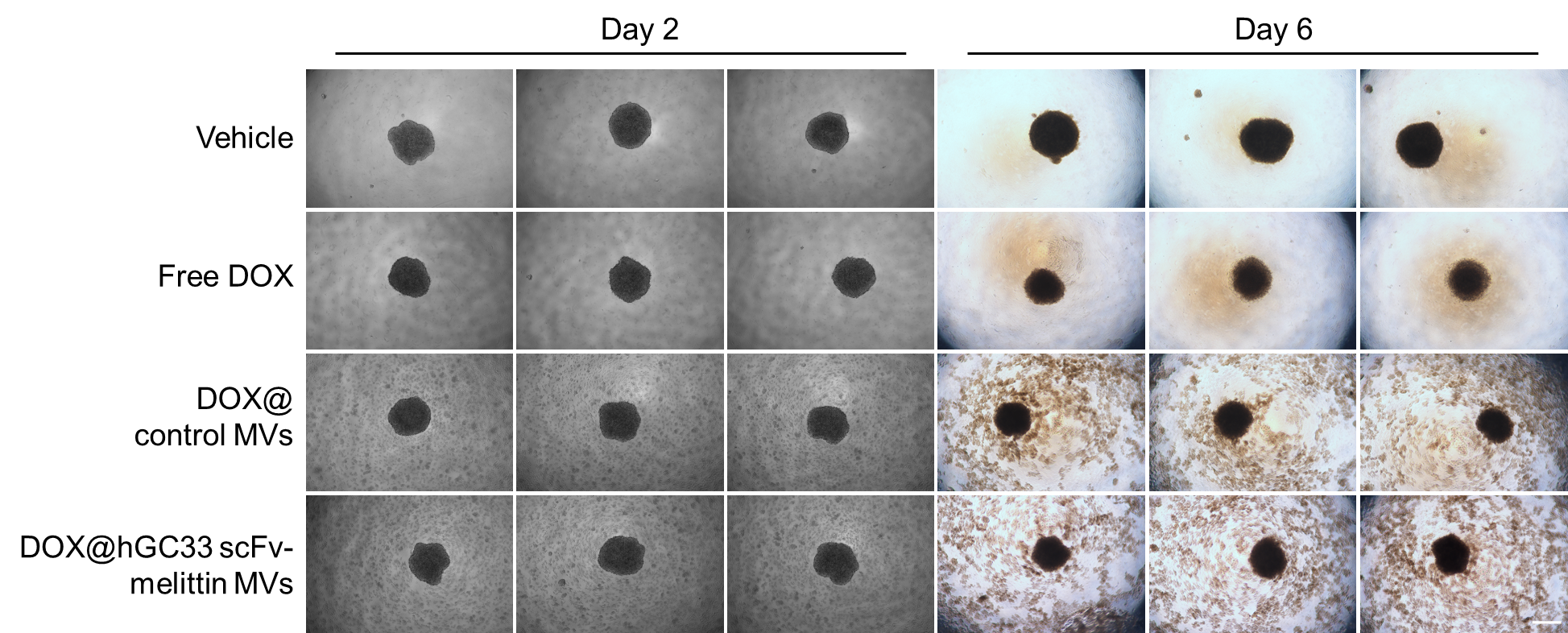
**

**Figure S18. Representative photograph of tumor spheroids treated with drugs at day 2 and day 6 post HepG2 cells seeding. Scale bar: 200 μm.**

**
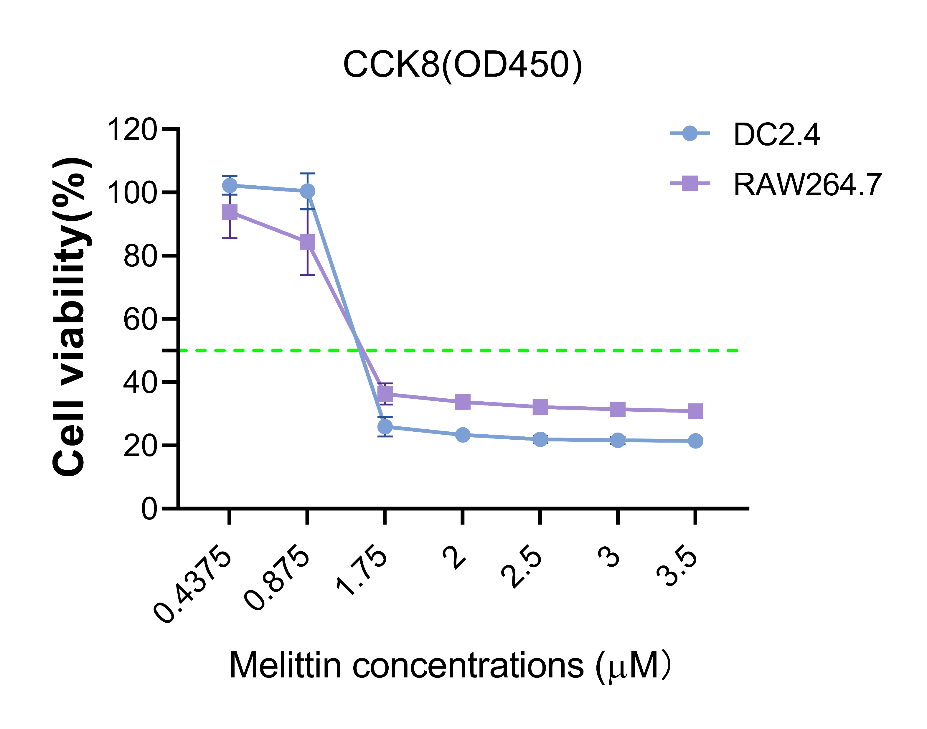
**

**Figure S19. Cell cytotoxicity test of free melittin in DC2.4 and RAW264.7.**

**
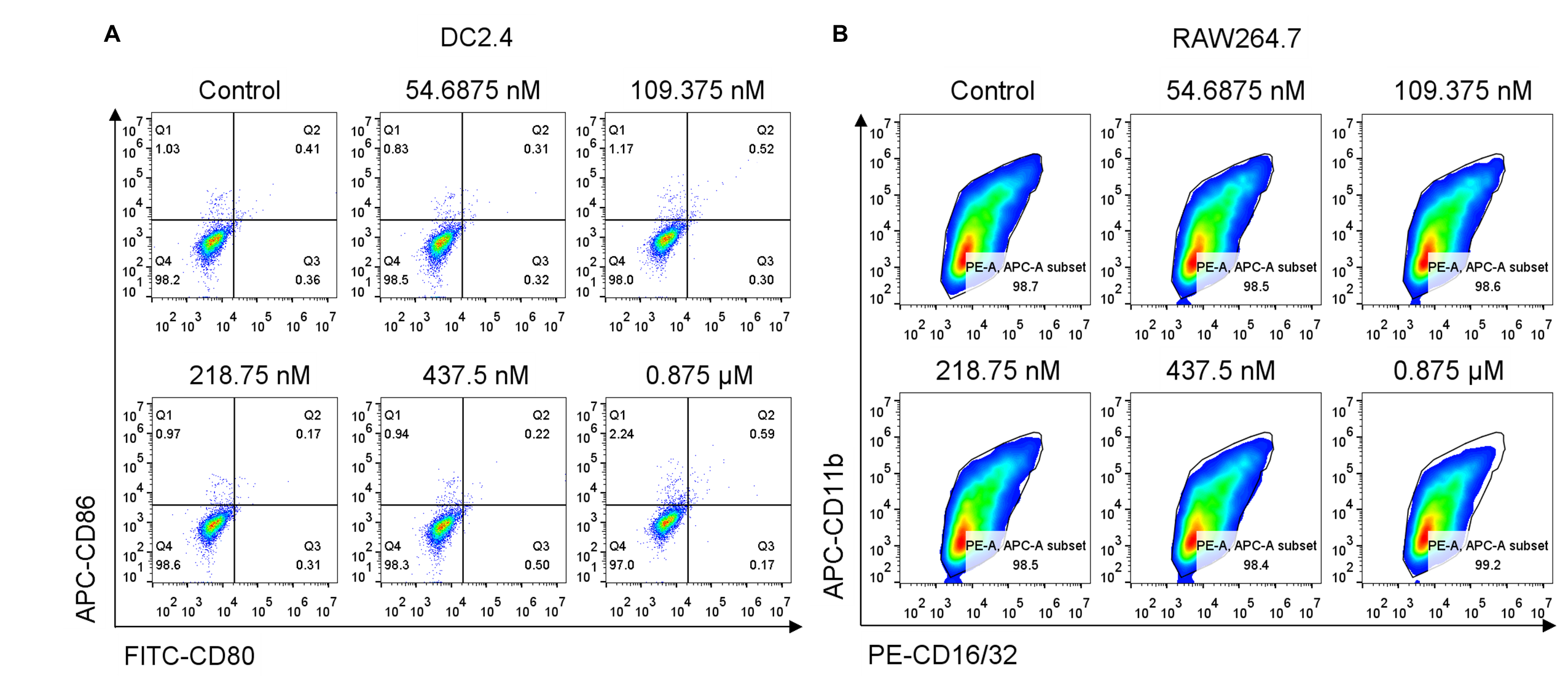
**

**Figure S20. Verification of the immunoregulatory effect of melittin. (A) Immunoregulatory effect of free melittin to DC2.4 and (B) RAW264.7 in different concentrations.**

**
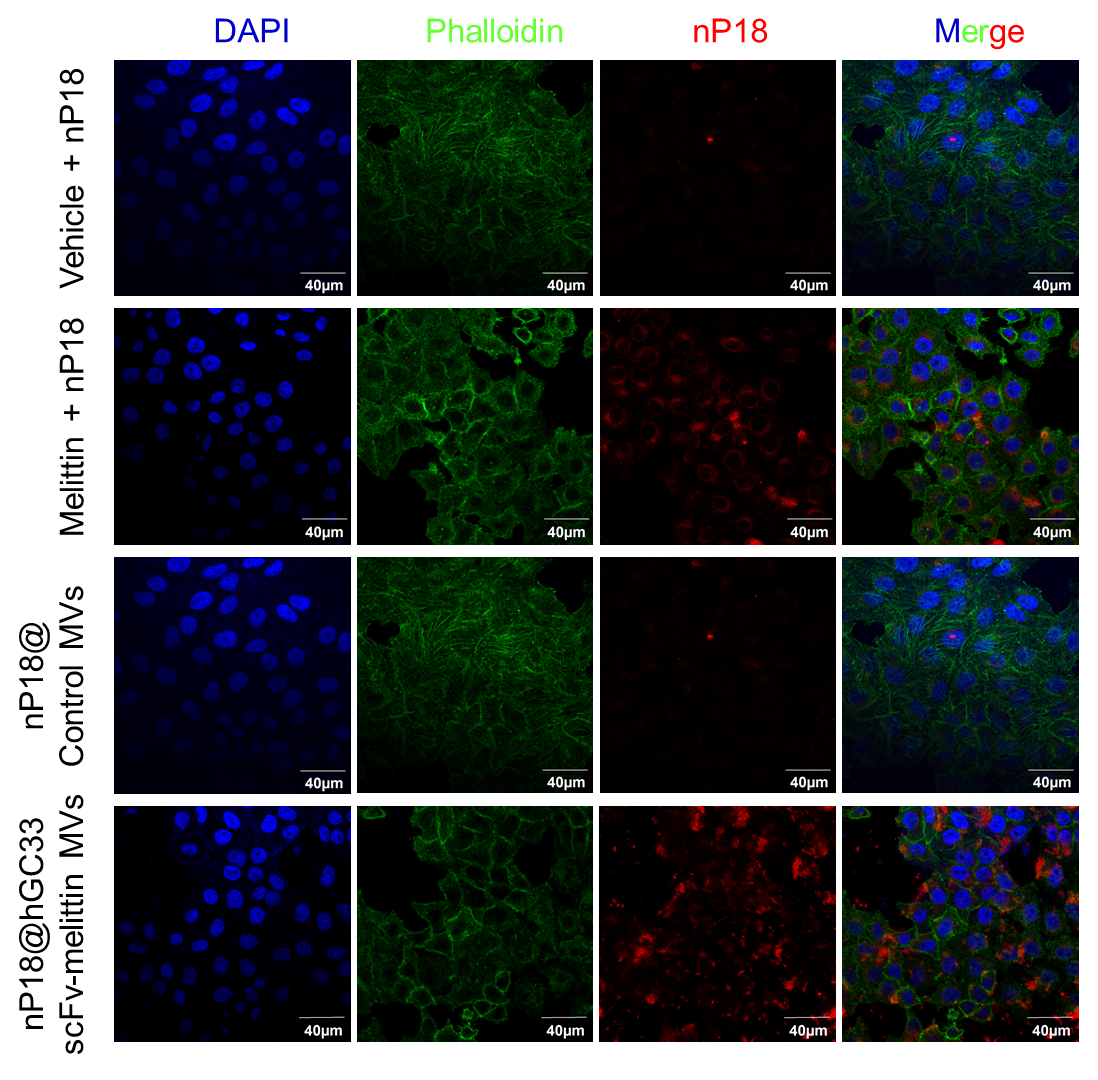
**

**Figure S21. nP18 uptaking by HepG2 in different treatments. Blue: DAPI, Green: phalloidin, Red: nP18. Scale bar: 40 μm.**

**
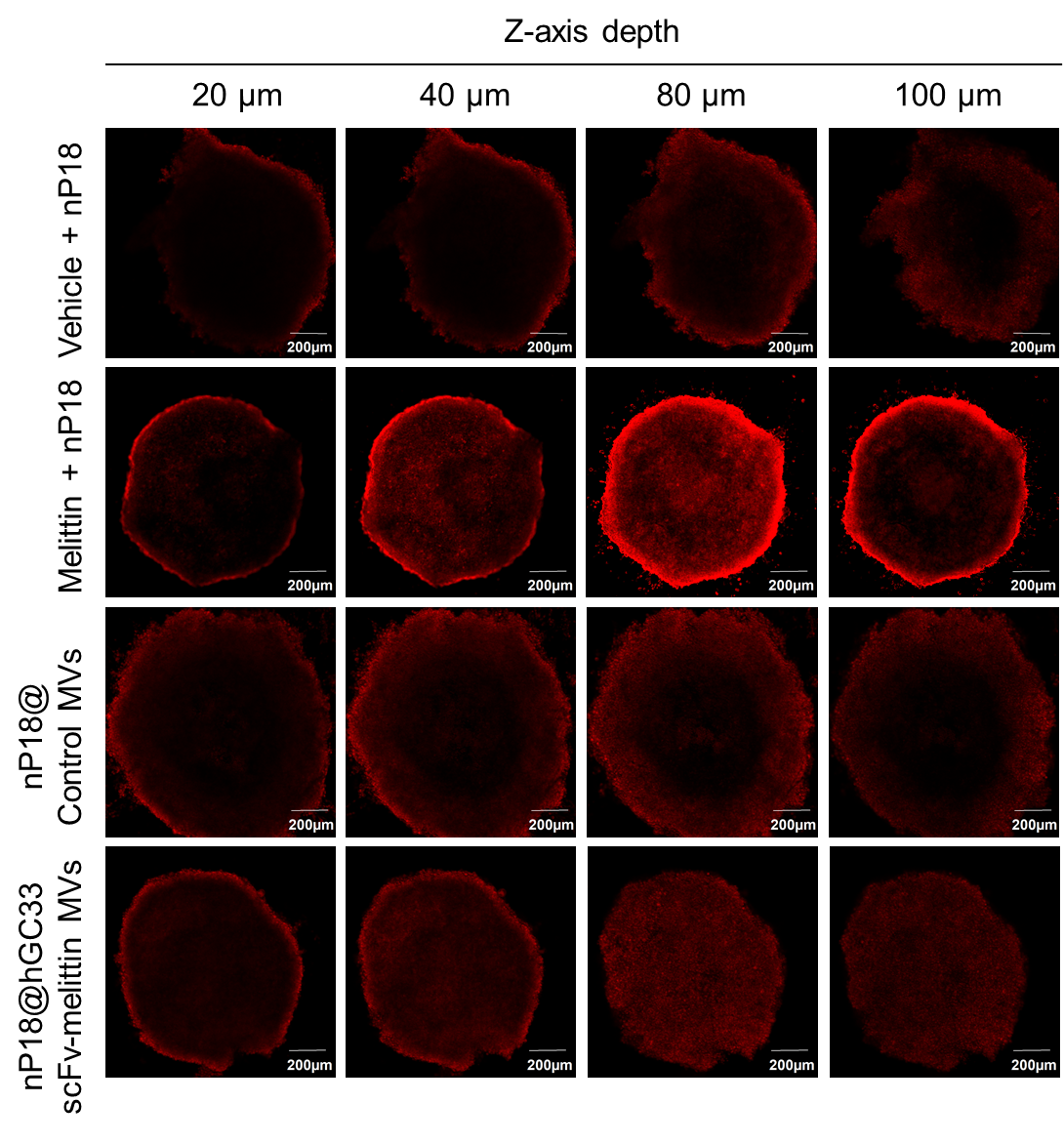
**

**Figure S22. nP18 uptaking by HepG2 multicellular tumor spheroid in different treatments. Red: nP18. Scale bar: 200 μm.**

**
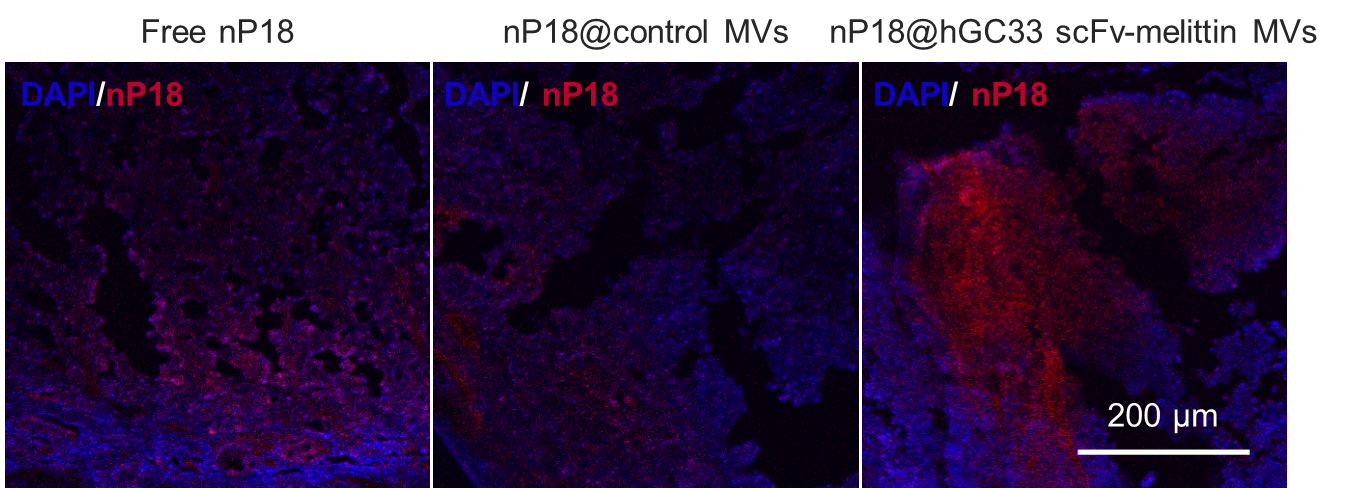
**

**Figure S23. Frozen sections of tumor tissue in different treatment groups.**

**
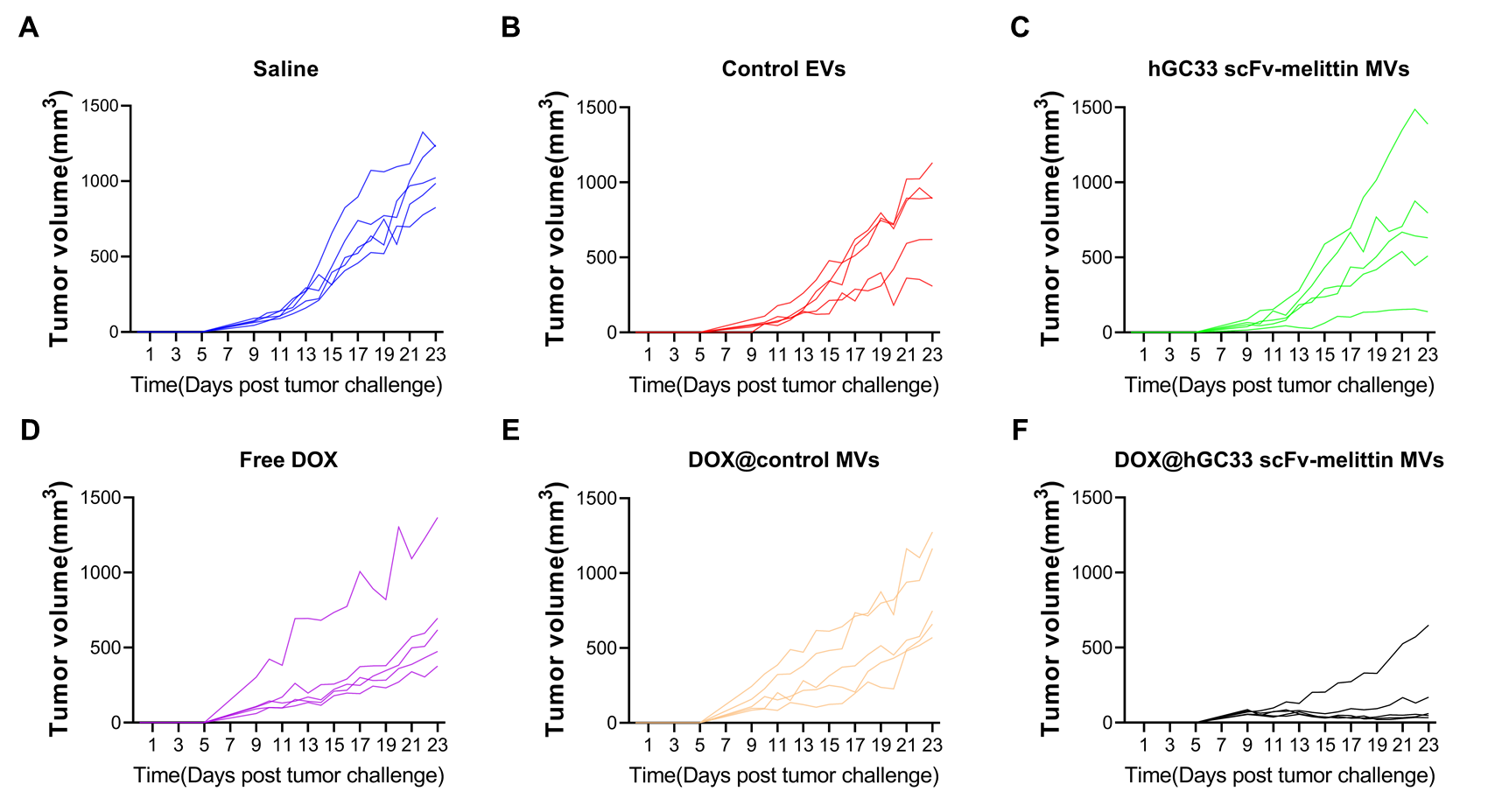
**

**Figure S24. (A)-(F) Tumor growth curve of each mouse in saline, control MVs, hGC33 scFv-melittin MVs, Free DOX, DOX@control MVs, and DOX@hGC33 scFv-melittin MVs treated groups, respectively.**


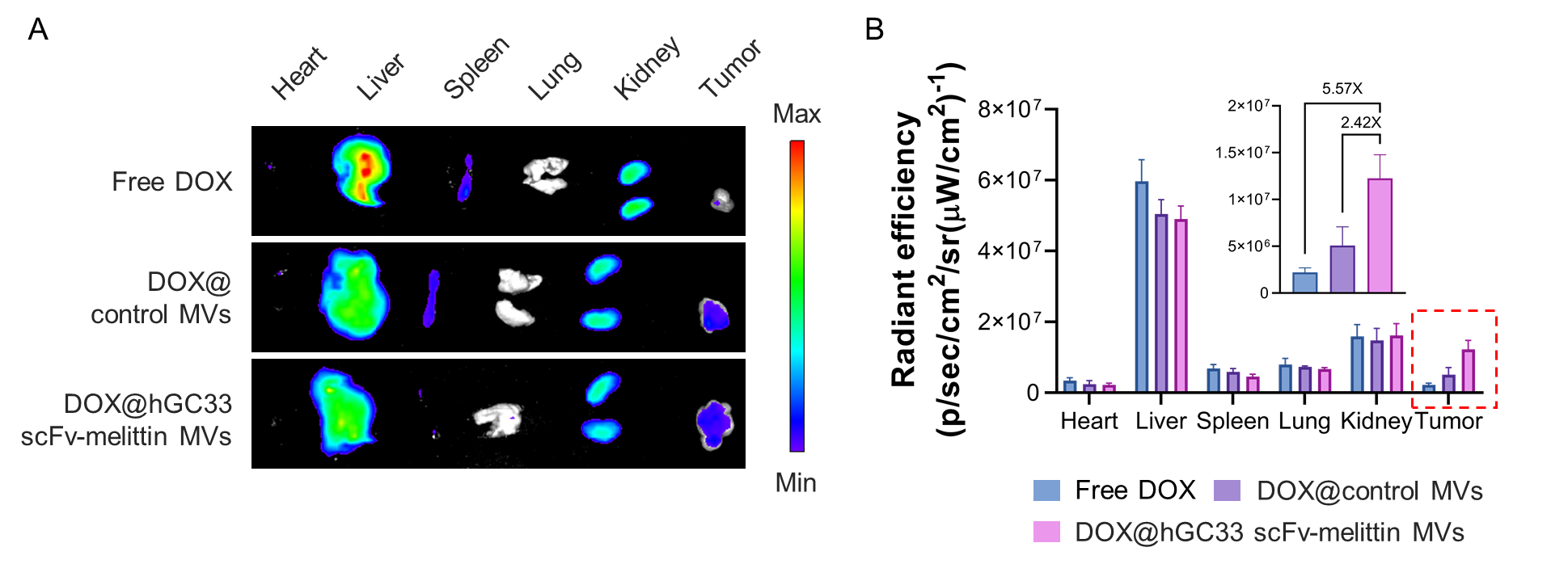


**Figure S25. Biodistribution of DOX in HepG2-bearing nude mice. (A) *Ex vivo* fluorescence imaging of major organs and tumors. (B) Relative fluorescence intensity of tumors and major organs after 12 h of injection (n = 3).**

**
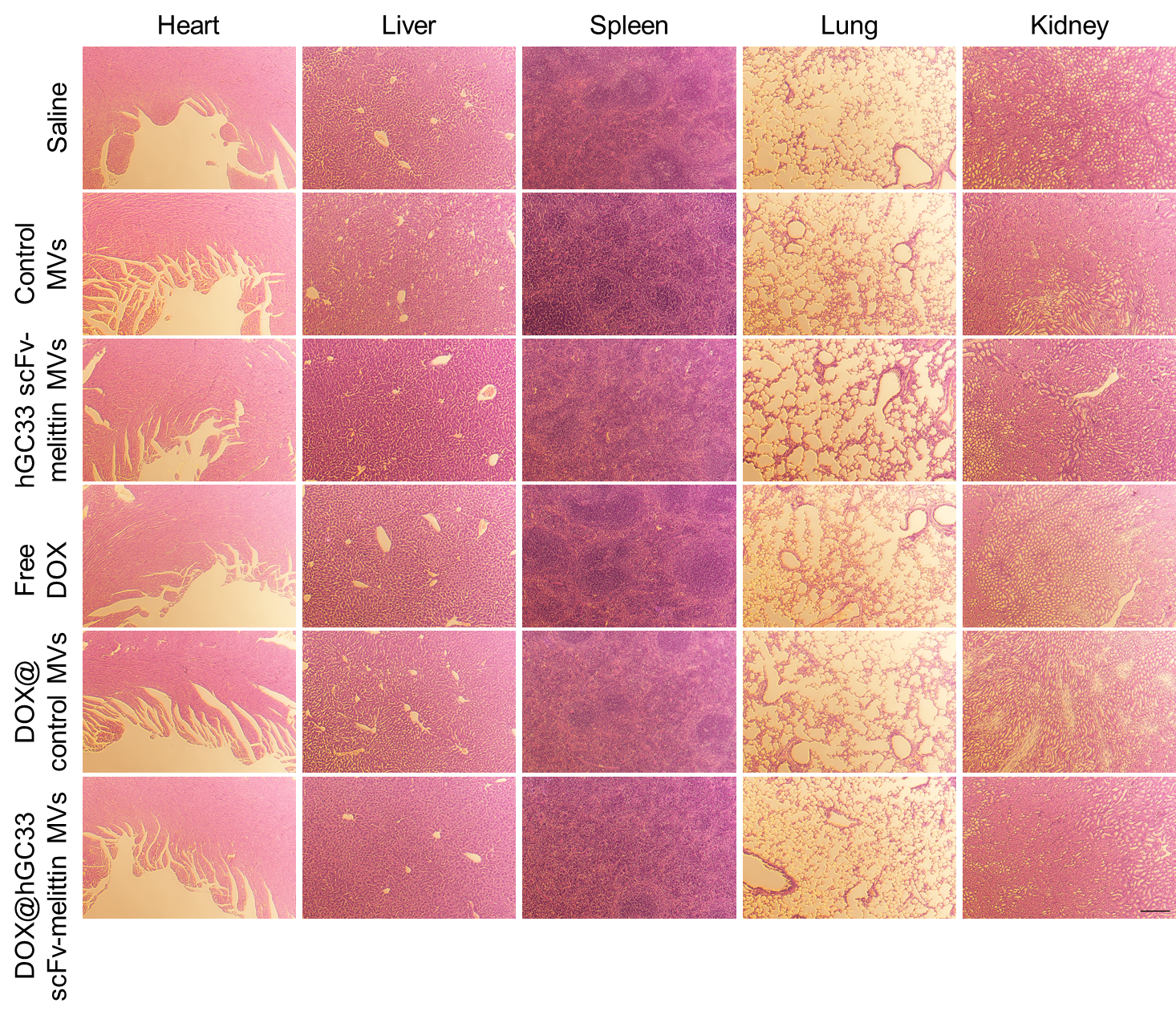
Figure S26. Hematoxylin-eosin (H & E) staining of heart, liver, spleen, lung, kidney of Balb/c nude mice from different treatment groups after sacrificed. Scale bar: 200 μm.**

**
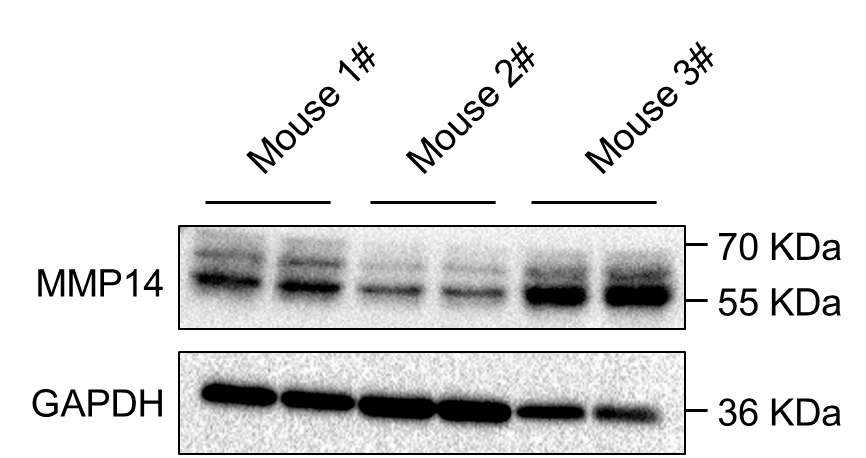
**

**Figure S27. Western blotting analysis of MMP14 expressing level in dissected tumor tissues from H22-GPC3 bearing Balb/c mice.**

**
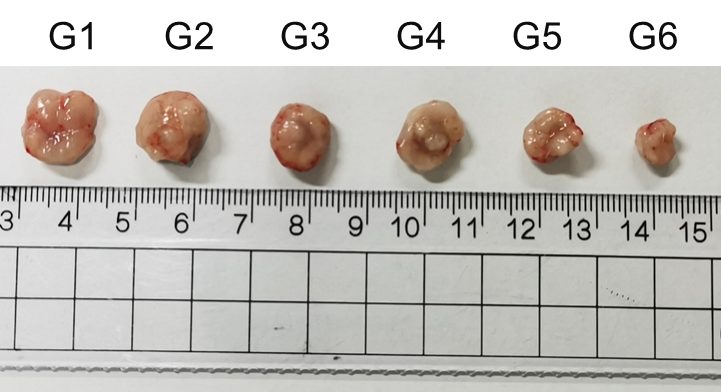
**

**Figure S28. Representative photograph of dissected tumors from H22-GPC3 bearing Balb/c mice. After sacrificed after 14 days of treatment, the resected tumors were photographed. Group 1, saline; Group 2, control MVs; Group 3, hGC33 scFv-melittin MVs; Group 4, free nP18 + US; Group 5, nP18@control MVs + US; Group 6, nP18@ hGC33 scFv-melittin MVs + US.**


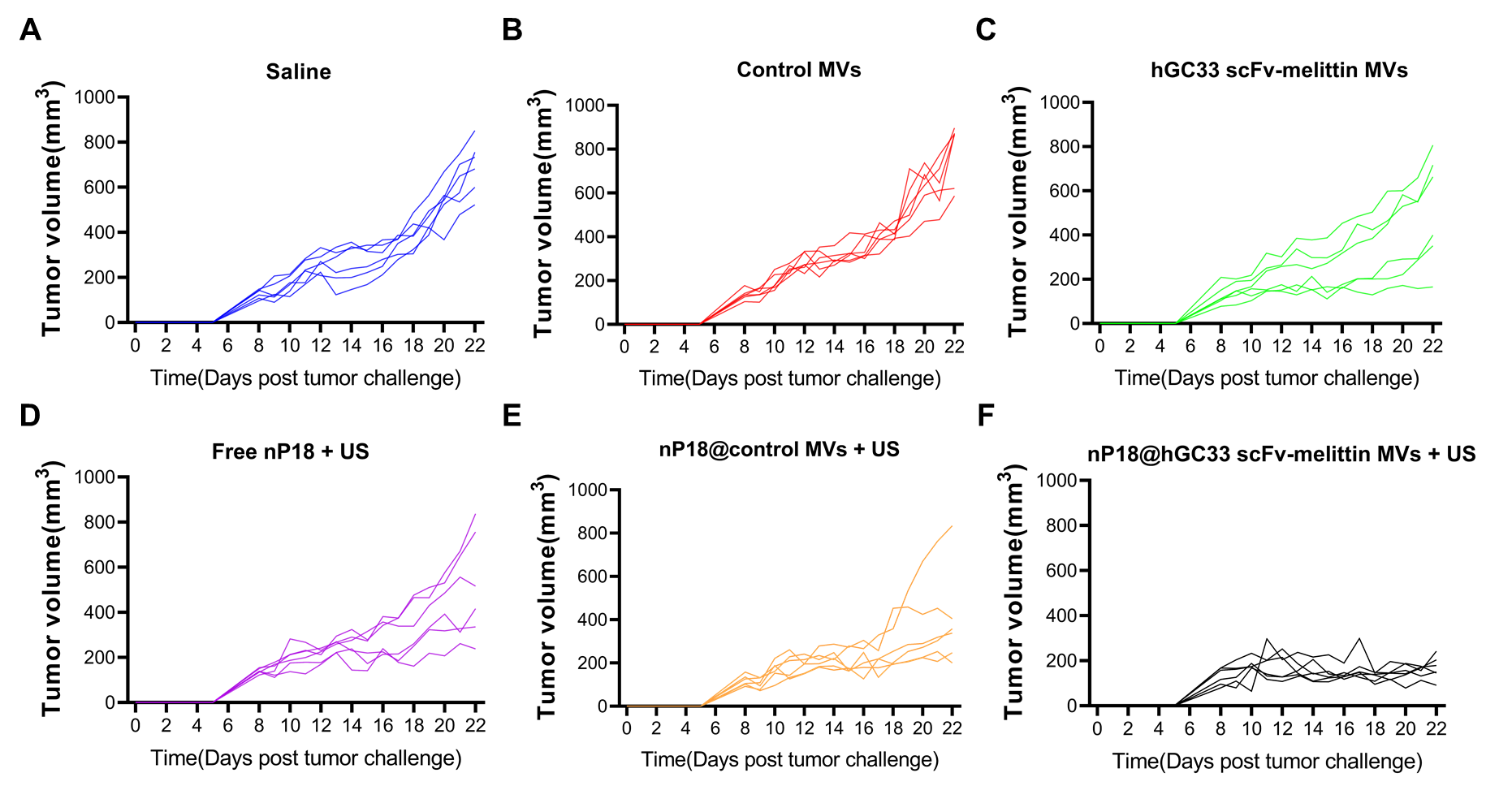


**Figure S29. (A)-(F) Tumor growth curve of each mouse in saline, control MVs, hGC33 scFv-melittin MVs, Free nP18 + US, nP18@control MVs + US, and nP18@hGC33 scFv-melittin MVs + US treated groups, respectively.**

**
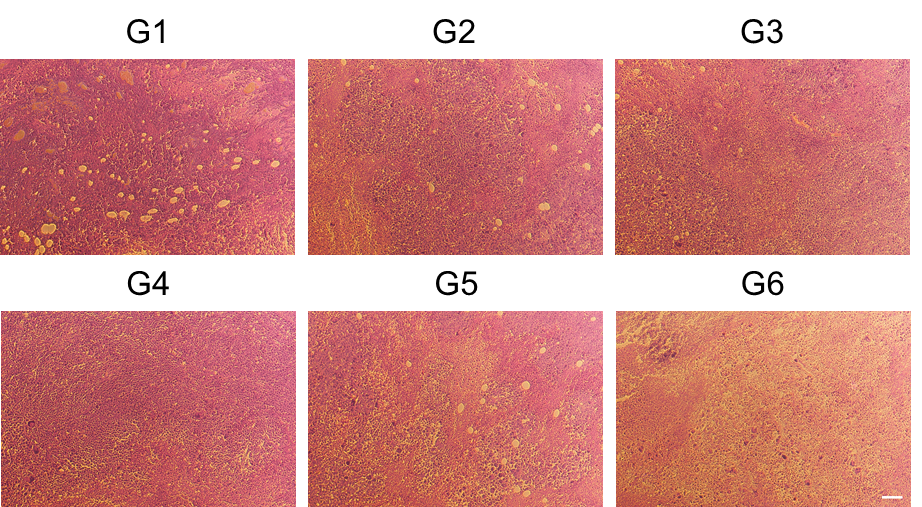
**

**Figure S30. Hematoxylin-eosin (H & E) staining of tumor tissues harvested from Balb/c mice treated with different treatments after sacrificed. Scale bar: 100 μm. Group 1, saline; Group 2, control MVs; Group 3, hGC33 scFv-melittin MVs; Group 4, free nP18 + US; Group 5, nP18@control MVs + US; Group 6, nP18@ hGC33 scFv-melittin MVs + US.**

**
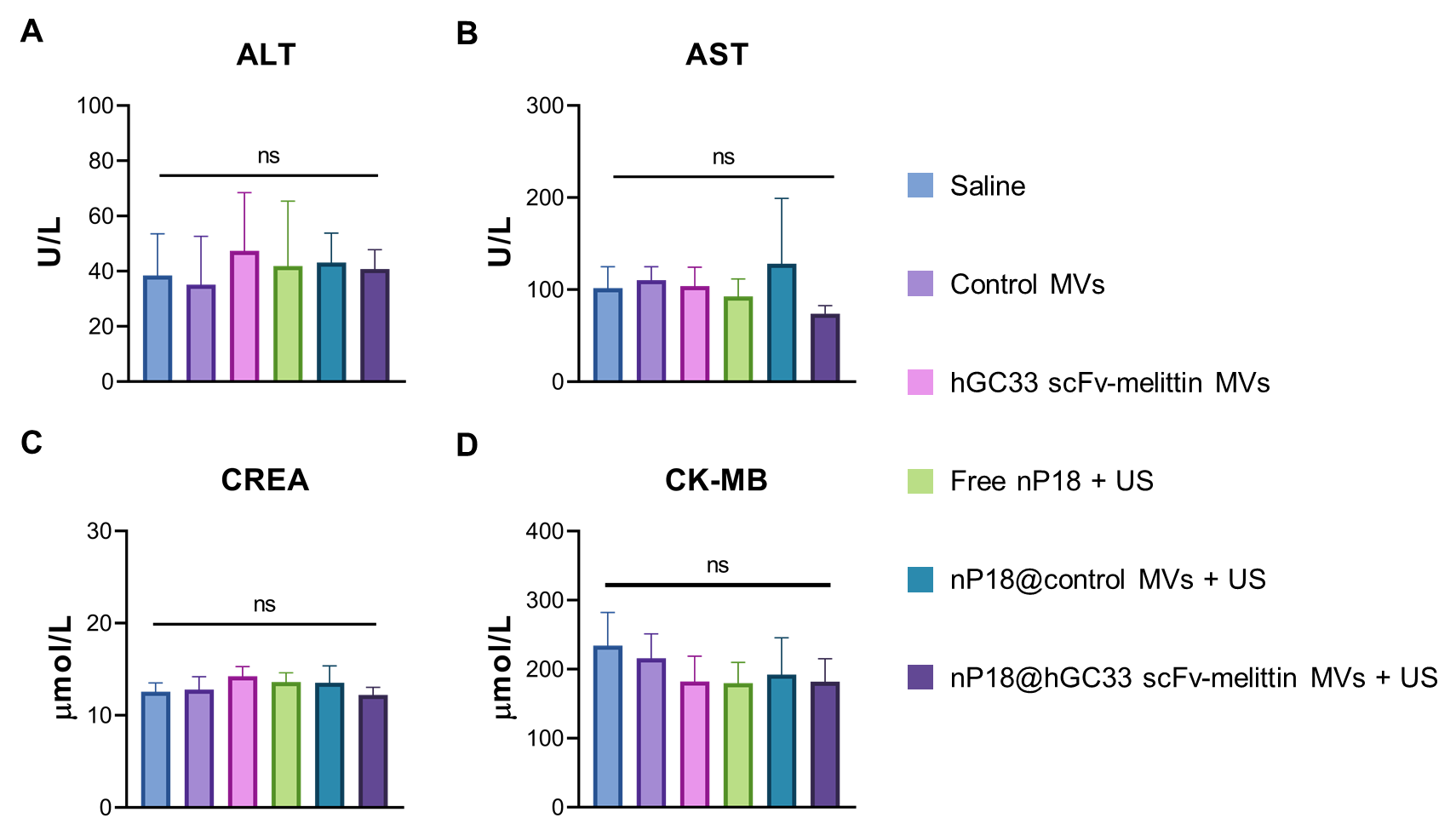
**

**Figure S31. Blood biochemical parameter. (A) Concentration of ALT (alanine transaminase) in different treatment groups. (B) Concentration of AST (aspartate transaminase) in different treatment groups. (C) Concentration of CK-MB (creatine kinase-MB) in different treatment groups. (D) Concentration of CREA (Creatinine) in different treatment groups. All the data are presented as means ± SD (n = 6). ns, not significant.**

**
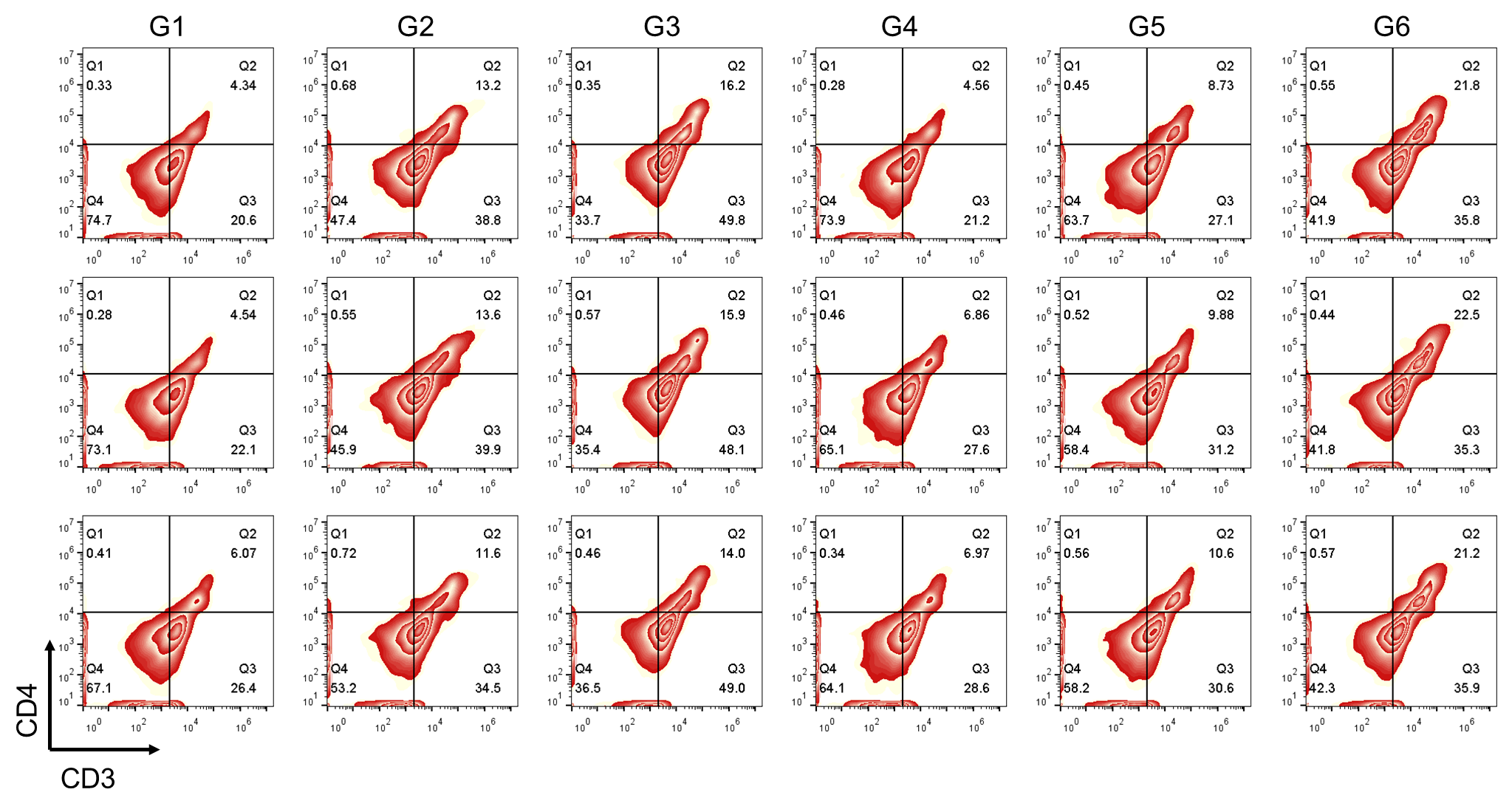
**

**Figure S32. Population of CD3^+^CD4^+^ T cells in the lymph node from H22-GPC3 bearing Balb/c mice. Group 1, saline; Group 2, control MVs; Group 3, hGC33 scFv-melittin MVs; Group 4, free nP18 + US; Group 5, nP18@control MVs + US; Group 6, nP18@ hGC33 scFv-melittin MVs + US.**

**
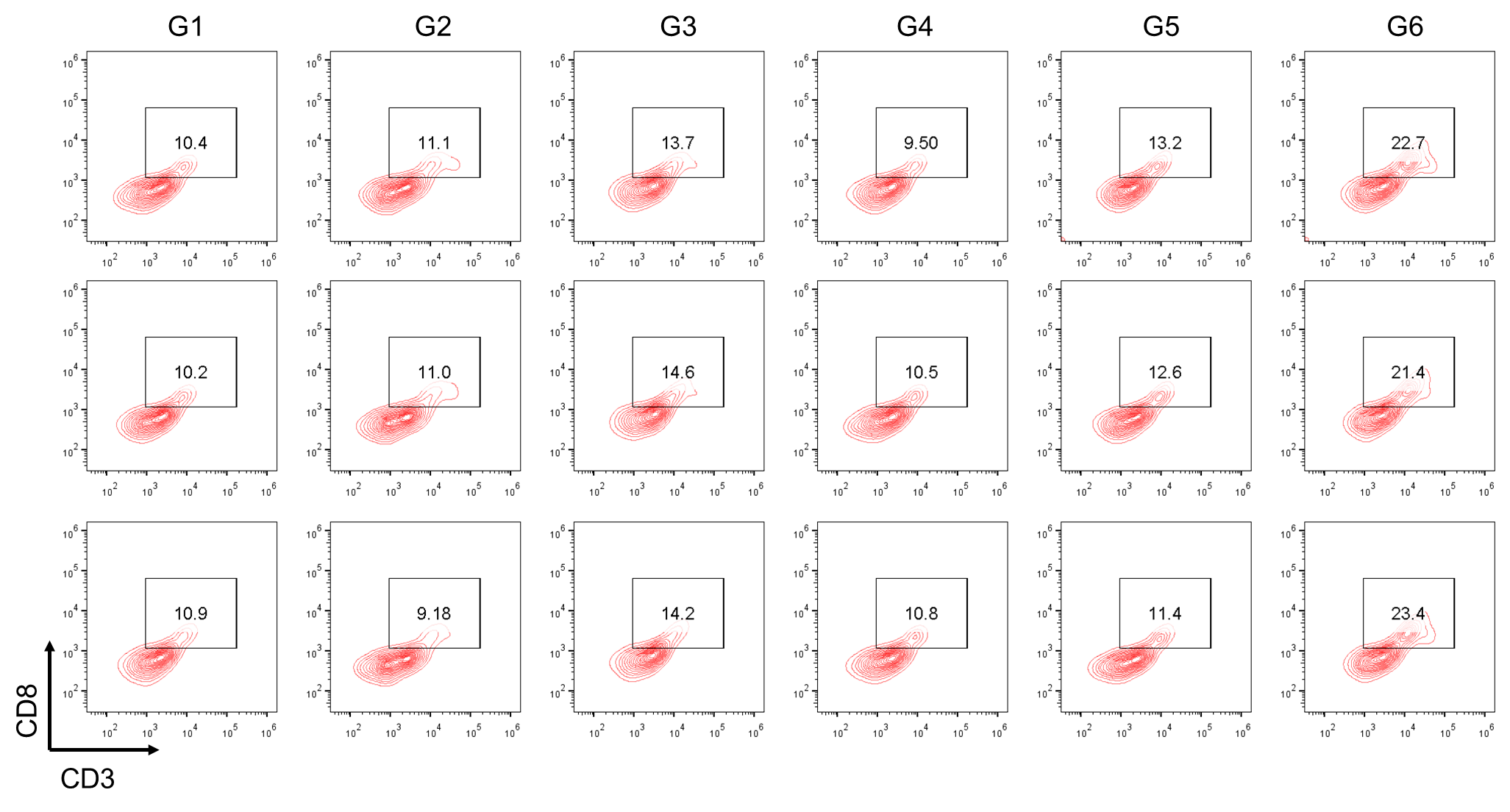
**

**Figure S33. Population of CD3^+^CD8^+^ T cells in the lymph node from H22-GPC3 bearing Balb/c mice. Group 1, saline; Group 2, control MVs; Group 3, hGC33 scFv-melittin MVs; Group 4, free nP18 + US; Group 5, nP18@control MVs + US; Group 6, nP18@ hGC33 scFv-melittin MVs + US.**

**
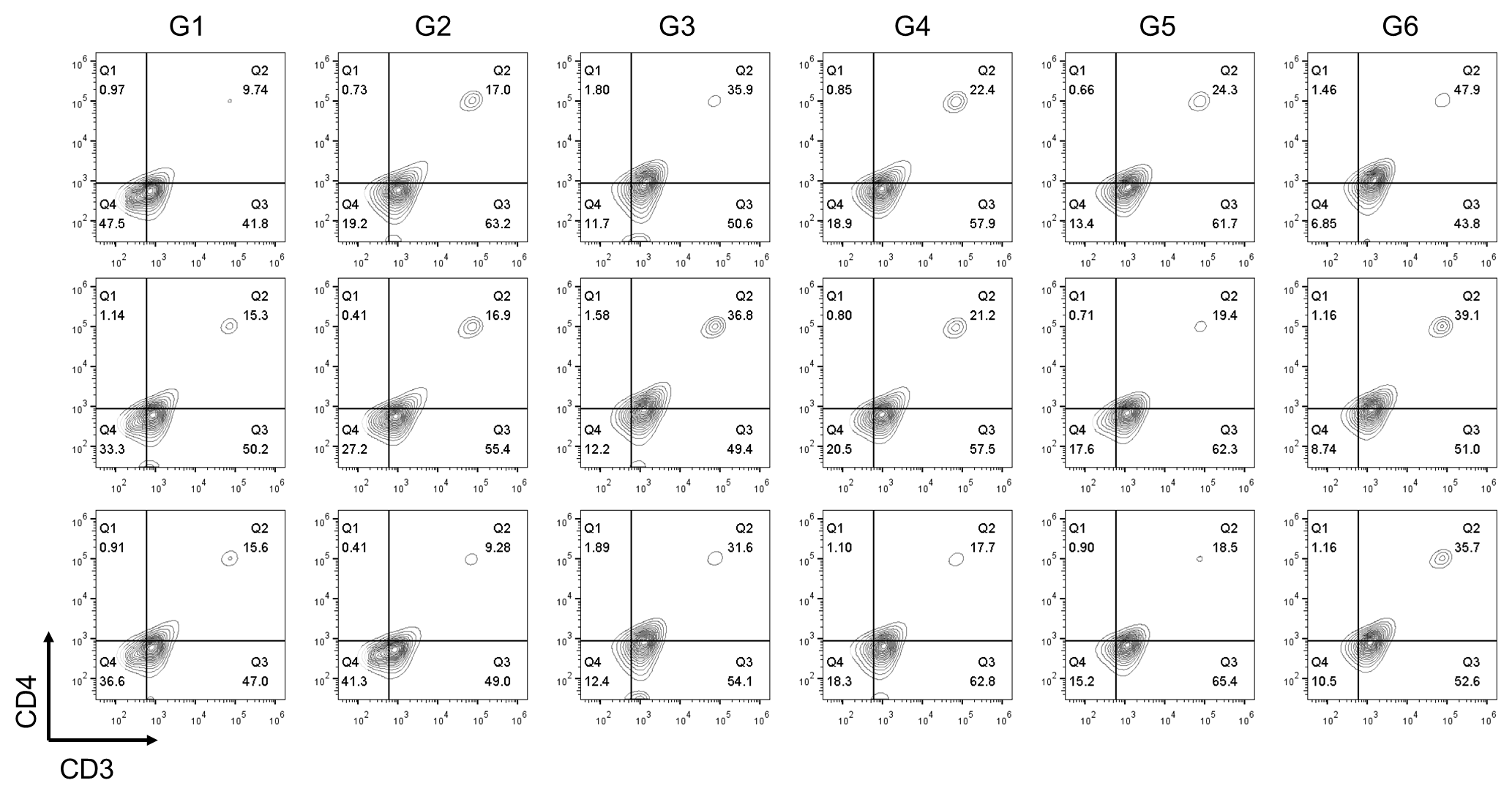
**

**Figure S34. Population of CD3^+^CD4^+^ T cells in the peripheral blood from H22-GPC3 bearing Balb/c mice. Group 1, saline; Group 2, control MVs; Group 3, hGC33 scFv-melittin MVs; Group 4, free nP18 + US; Group 5, nP18@control MVs + US; Group 6, nP18@ hGC33 scFv-melittin MVs + US.**

**
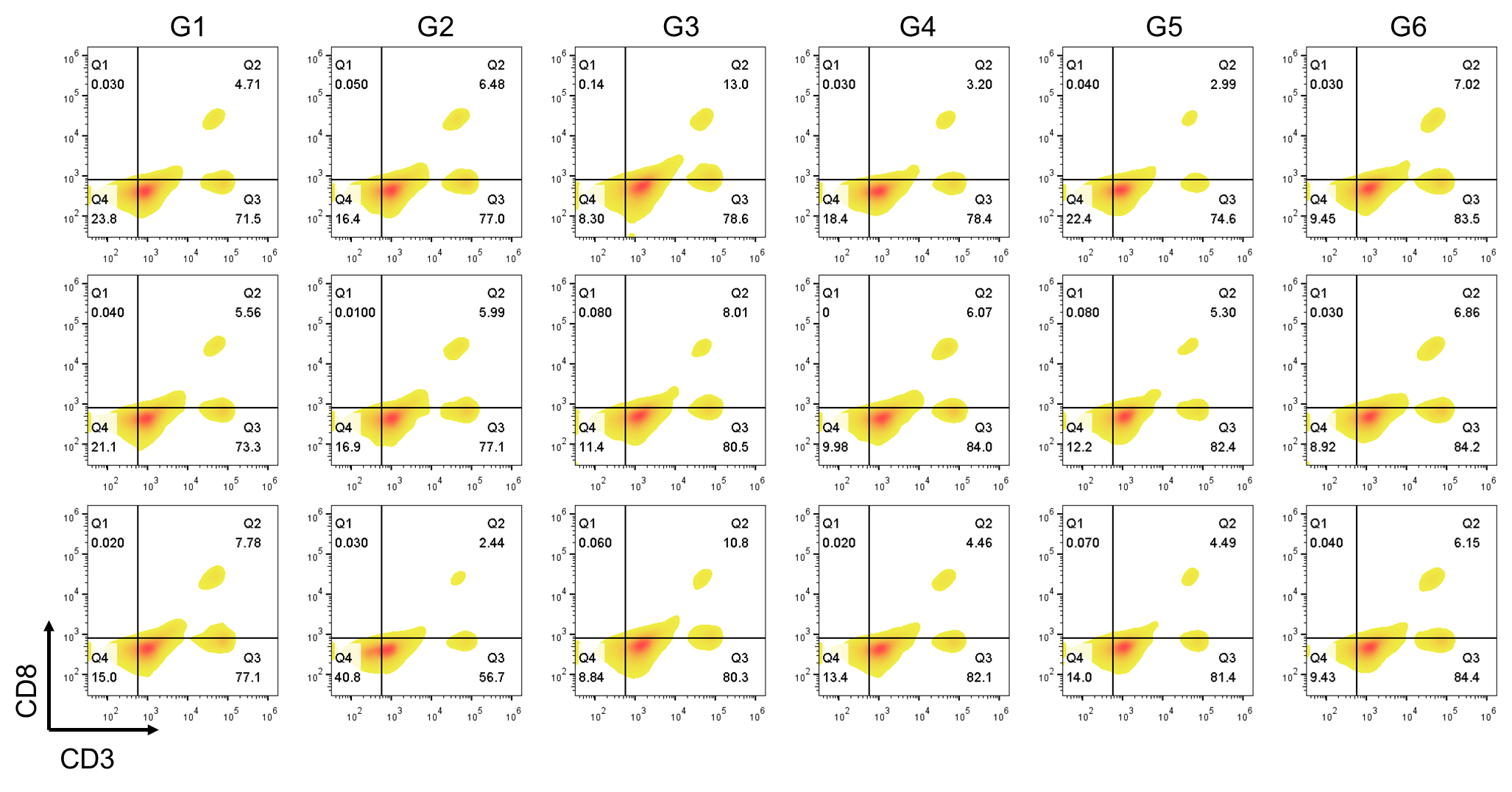
**

**Figure S35. Population of CD3^+^CD8^+^ T cells in the peripheral blood from H22-GPC3 bearing Balb/c mice. Group 1, saline; Group 2, control MVs; Group 3, hGC33 scFv-melittin MVs; Group 4, free nP18 + US; Group 5, nP18@control MVs + US; Group 6, nP18@ hGC33 scFv-melittin MVs + US.**

**
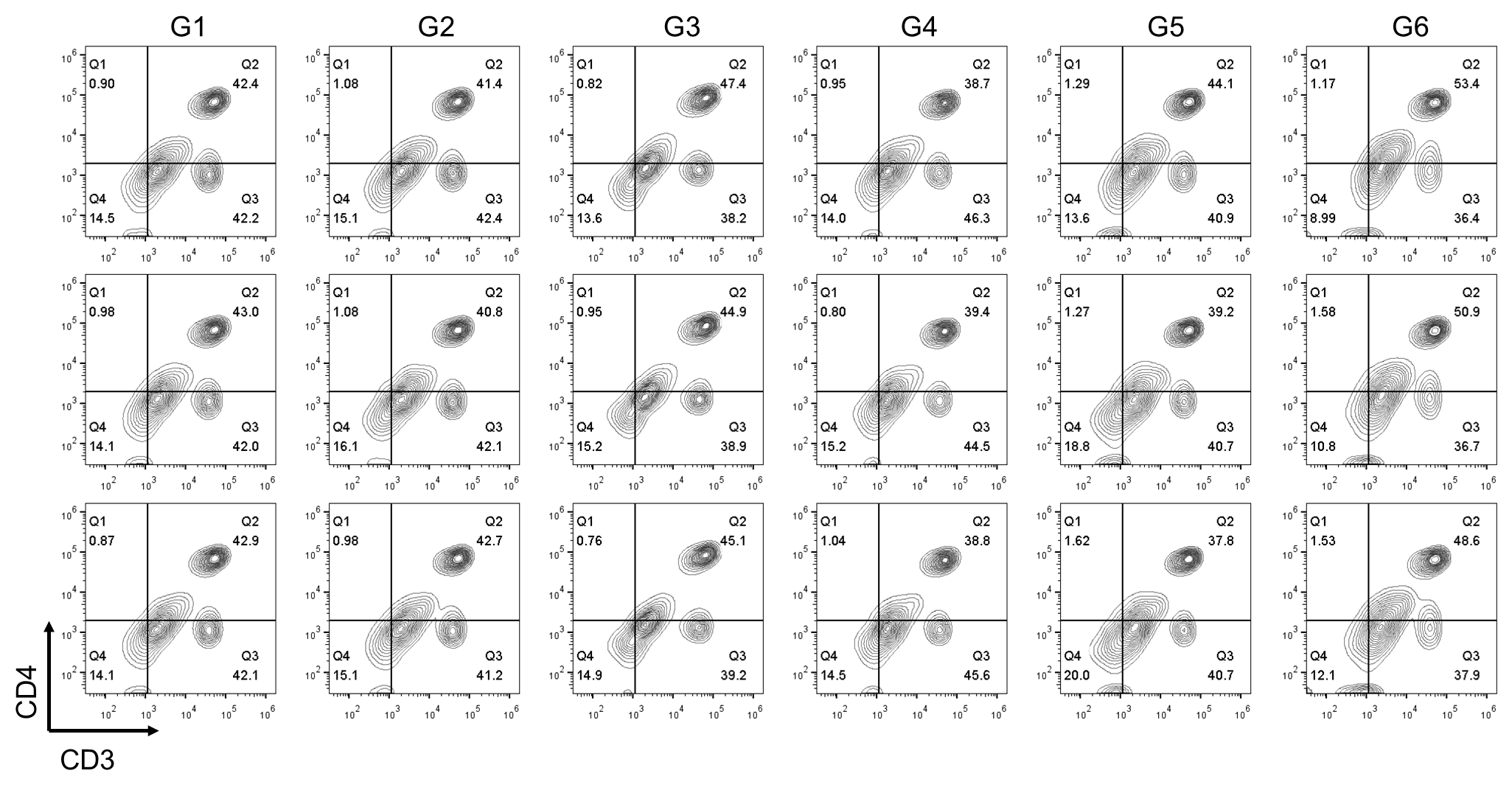
**

**Figure S36. Population of CD3^+^CD4^+^ T cells in the spleen from H22-GPC3 bearing Balb/c mice. Group 1, saline; Group 2, control MVs; Group 3, hGC33 scFv-melittin MVs; Group 4, free nP18 + US; Group 5, nP18@control MVs + US; Group 6, nP18@ hGC33 scFv-melittin MVs + US.**

**
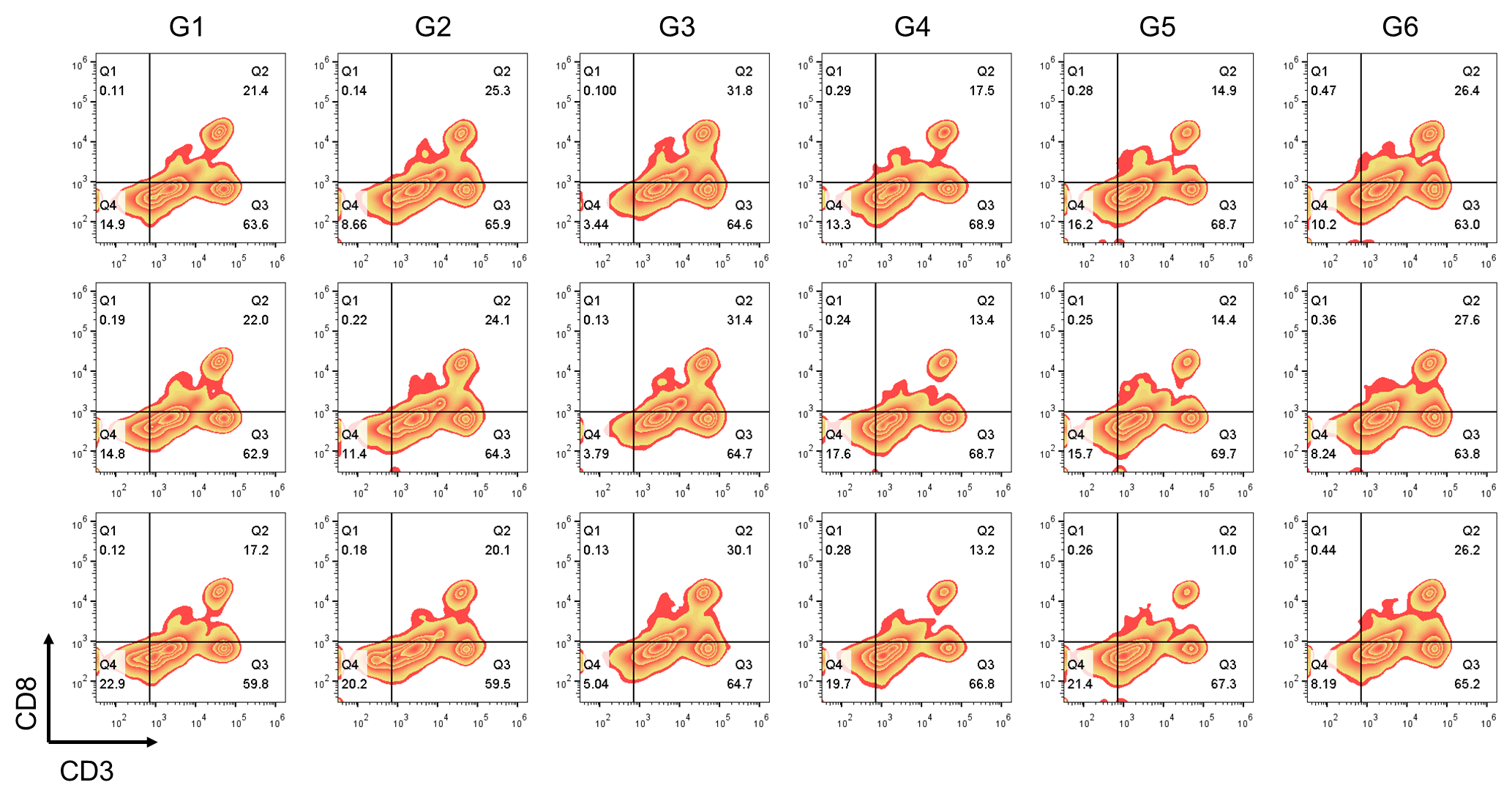
**

**Figure S37. Population of CD3^+^CD8^+^ T cells in the spleen from H22-GPC3 bearing Balb/c mice. Group 1, saline; Group 2, control MVs; Group 3, hGC33 scFv-melittin MVs; Group 4, free nP18 + US; Group 5, nP18@control MVs + US; Group 6, nP18@ hGC33 scFv-melittin MVs + US.**

**
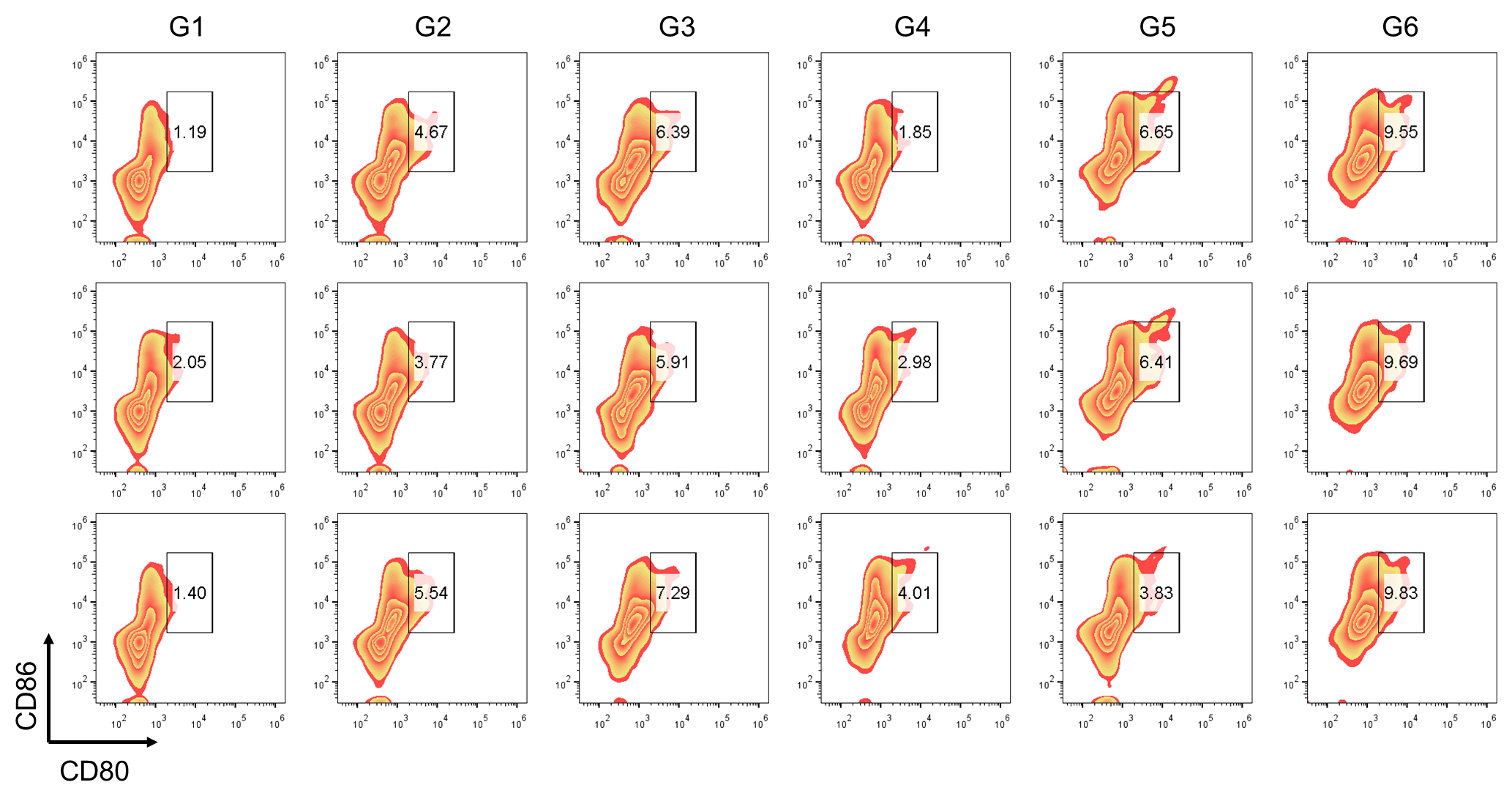
**

**Figure S38. Population of CD80^+^CD6^+^ DCs in the spleen from H22-GPC3 bearing Balb/c mice. Group 1, saline; Group 2, control MVs; Group 3, hGC33 scFv-melittin MVs; Group 4, free nP18 + US; Group 5, nP18@control MVs + US; Group 6, nP18@ hGC33 scFv-melittin MVs + US.**

**
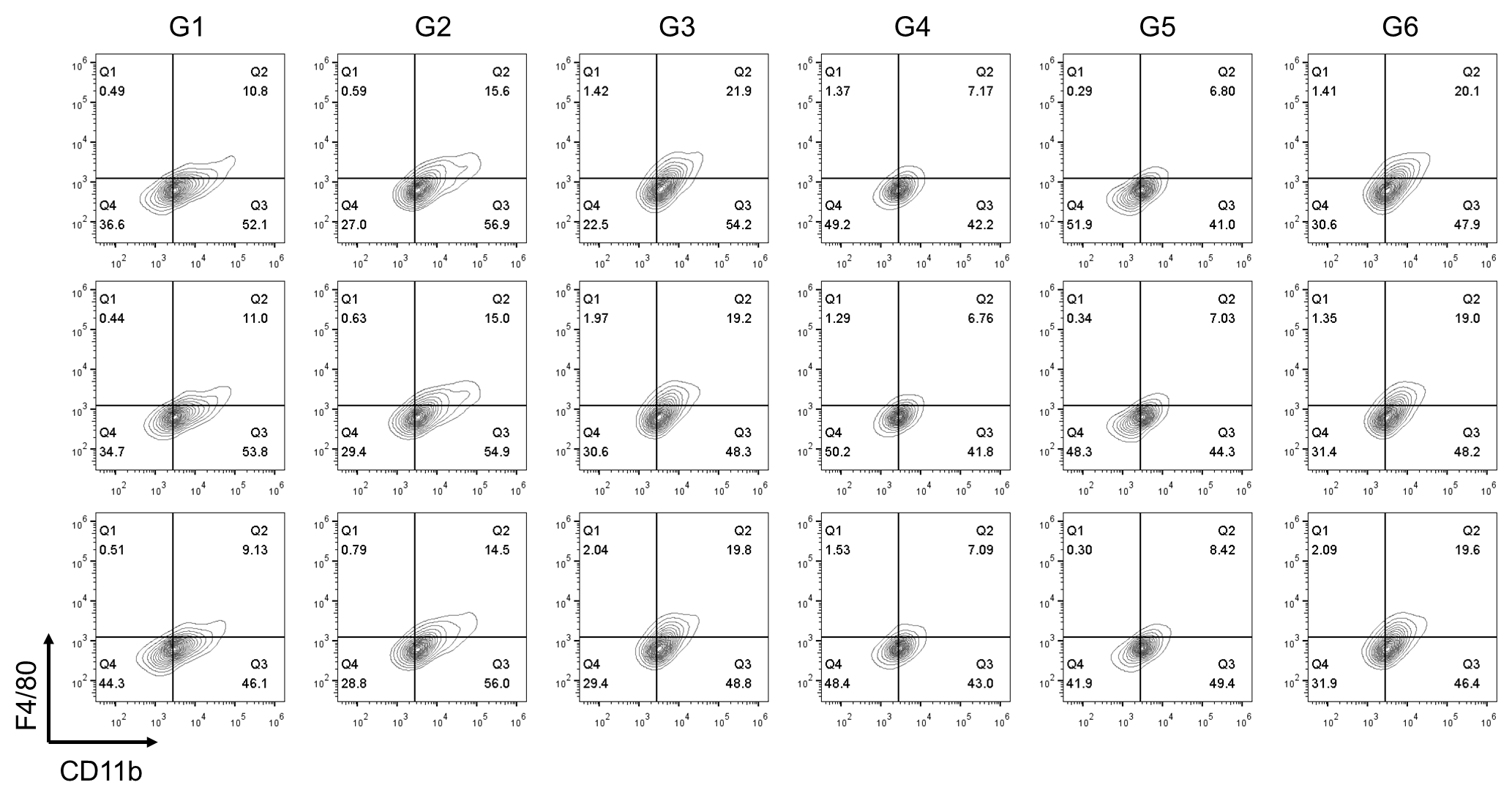
**

**Figure S39. Population of CD11b^+^F4/80^+^ macrophages in the spleen from H22-GPC3 bearing Balb/c mice. Group 1, saline; Group 2, control MVs; Group 3, hGC33 scFv-melittin MVs; Group 4, free nP18 + US; Group 5, nP18@control MVs + US; Group 6, nP18@ hGC33 scFv-melittin MVs + US.**
